# M2-like tumor-associated macrophage-related biomarkers to construct a novel prognostic signature, reveal the immune landscape, and screen drugs in hepatocellular carcinoma

**DOI:** 10.3389/fimmu.2022.994019

**Published:** 2022-09-13

**Authors:** Xiaodong Qu, Xingyu Zhao, Kexin Lin, Na Wang, Xuezhi Li, Songbo Li, Luyao Zhang, Yongquan Shi

**Affiliations:** State Key Laboratory of Cancer Biology, National Clinical Research Center for Digestive Diseases, Xijing Hospital, Fourth Military Medical University, Xi’an, China

**Keywords:** hepatocellular carcinoma, tumor-associated macrophages, prognostic signature, immune landscape, drug screening

## Abstract

**Background:**

M2-like tumor-associated macrophages (M2-like TAMs) have important roles in the progression and therapeutics of cancers. We aimed to detect novel M2-like TAM-related biomarkers in hepatocellular carcinoma (HCC) *via* integrative analysis of single-cell RNA-seq (scRNA-seq) and bulk RNA-seq data to construct a novel prognostic signature, reveal the “immune landscape”, and screen drugs in HCC.

**Methods:**

M2-like TAM-related genes were obtained by overlapping the marker genes of TAM identified from scRNA-seq data and M2 macrophage modular genes identified by weighted gene co-expression network analysis (WGCNA) using bulk RNA-seq data. Univariate Cox regression and least absolute shrinkage and selection operator (LASSO) regression analyses were carried out to screen prognostic genes from M2-like TAM-related genes, followed by a construction of a prognostic signature, delineation of risk groups, and external validation of the prognostic signature. Analyses of immune cells, immune function, immune evasion scores, and immune-checkpoint genes between high- and low-risk groups were done to further reveal the immune landscape of HCC patients. To screen potential HCC therapeutic agents, analyses of gene–drug correlation and sensitivity to anti-cancer drugs were conducted.

**Results:**

A total of 127 M2-like TAM-related genes were identified by integrative analysis of scRNA-seq and bulk-seq data. PDLIM3, PAM, PDLIM7, FSCN1, DPYSL2, ARID5B, LGALS3, and KLF2 were screened as prognostic genes in HCC by univariate Cox regression and LASSO regression analyses. Then, a prognostic signature was constructed and validated based on those genes for predicting the survival of HCC patients. In terms of drug screening, expression of PAM and LGALS3 was correlated positively with sensitivity to simvastatin and ARRY-162, respectively. Based on risk grouping, we predicted 10 anticancer drugs with high sensitivity in the high-risk group, with epothilone B having the lowest half-maximal inhibitory concentration among all drugs tested.

**Conclusions:**

Our findings enhance understanding of the M2-like TAM-related molecular mechanisms involved in HCC, reveal the immune landscape of HCC, and provide potential targets for HCC treatment.

## Introduction

Primary liver cancer is the third most deadly malignancy worldwide. It accounted for ~906,000 new cases and ~830,000 deaths in 2020, with hepatocellular carcinoma (HCC) accounting for 75–85% of cases (1). The overall burden of HCC worldwide has increased over time (2). In the USA, the incidence of HCC has tripled in the last three decades (3). The median survival and 5-year survival for patients with HCC after primary hepatic resection are 47 months and 45%, respectively. However, HCC recurs in 54% of patients, resulting in a 24% reduction in 5-year survival and a 54-month reduction in median survival (4). HCC pathogenesis is incompletely understood and the prognosis is not promising. Hence, there is a need for more in-depth research and identification of innovative “signatures” to predict the prognosis of HCC patients.

The tumor microenvironment (TME) consists mainly of tumor cells, immune cells, and inflammatory cells (5). Among them, tumor-associated macrophages (TAMs) play an important part in tumor progression. Macrophages can be polarized into M1 and M2 types. TAMs are not present in the steady state of an organism but are observed in several types of tumors. Therefore, TAMs are not always considered an additional subpopulation of macrophages. TAMs share the characteristic polarization of M1 and M2 macrophages (6), but their function is similar to that of M2 macrophages (i.e., M2-like TAMs). TAMs promote cancer angiogenesis by producing matrix metalloproteinases, cathepsins, and angiogenic growth factors (7, 8). In addition, TAMs facilitate tumor metastasis by promoting epithelial–mesenchymal transition (9). More importantly, TAM can interact with multiple types of immune cells within the TME. They can suppress cluster of differentiation (CD)8^+^ T cells, induce dysfunction of natural killer (NK) cells and NK T cells, and suppress effector T cells indirectly by amplifying T regulatory cells (T_regs_), thereby reducing the number of anti-tumor immune cells to accelerate tumorigenesis (10). Therefore, in-depth investigation of the role of M2-like TAMs in HCC development and constructing a prognostic signature associated with M2-like TAMs are very important and rational approaches.

**Figure 1:**
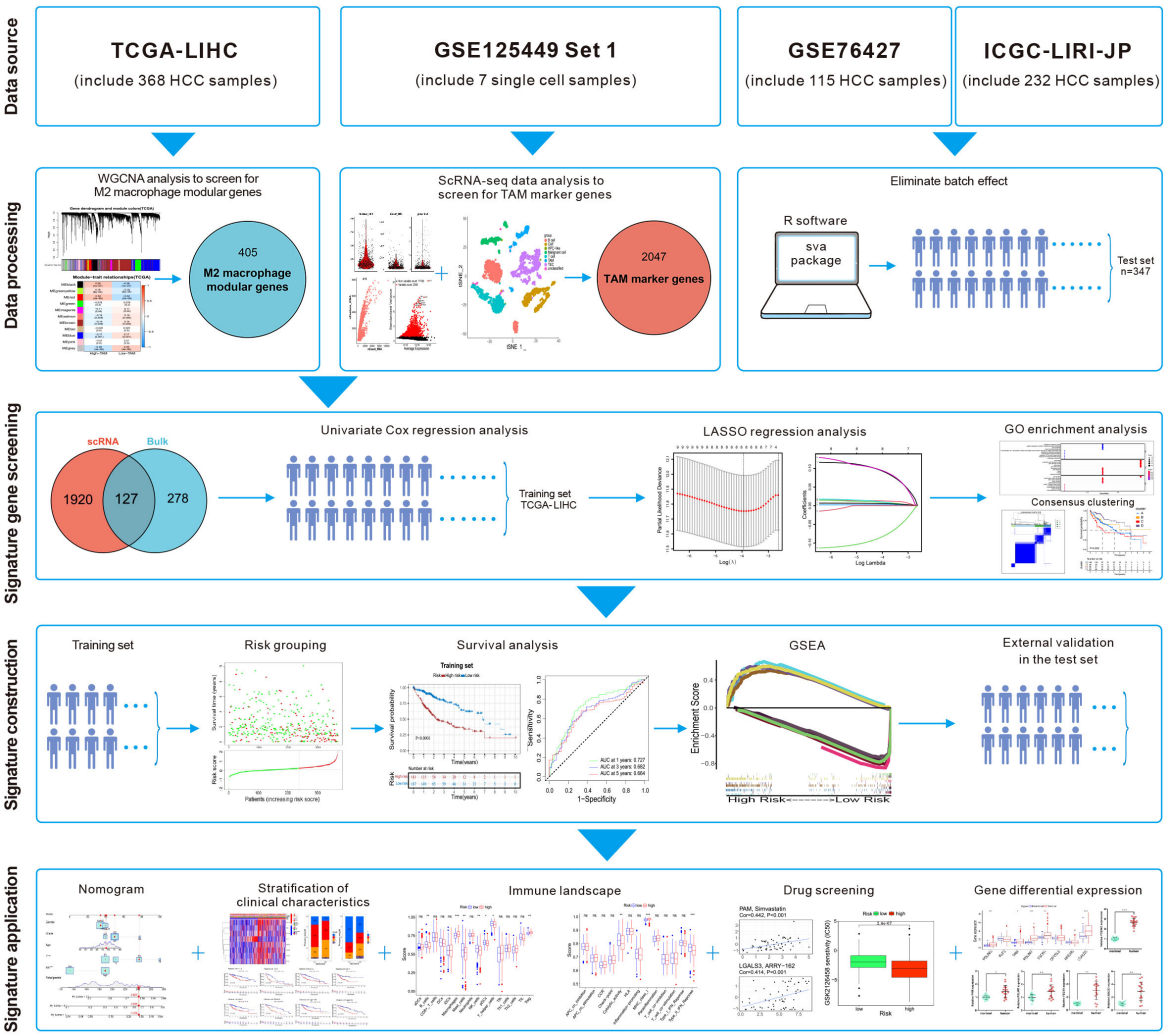
Flowchart of this study.

Single-cell RNA-sequencing (scRNA-seq) enables study of the heterogeneity within tumors at the cellular level (11). Ma et al. undertook scRNA-seq on liver-cancer specimens (9 HCC and 10 intrahepatic cholangiocarcinomas) (12). They carried out bioinformatics analysis to screen for marker genes. We combined the scRNA-seq data with The Cancer Genome Atlas-Liver Hepatocellular Carcinoma (TCGA-LIHC) dataset. Then, eight M2-like TAM-related prognostic genes were identified and a novel prognostic signature of HCC was constructed. After validation in the test set, this M2-like TAM-related signature was found to predict the prognosis of patients with HCC. Differences in “immune landscapes” and immunotherapy based on risk grouping were revealed and potential anticancer drugs predicted. The flowchart of this study is illustrated in **Figure 1**.

## Materials and methods

### Acquisition and processing of data

The GSE125449 single-cell transcriptome profiles of liver cancer was downloaded from the Gene Expression Omnibus (GEO) database (www.ncbi.nlm.nih.gov/). We selected seven HCC samples from Set 1 for analyses. The “Seurat” package (13) was used for processing scRNA-seq data, including data filtering (cells and genes), normalization, principal component analysis (PCA), and t-distributed stochastic neighbor embedding (t-SNE). The quality control standards referred to the uploader (12). Cell samples with >20% mitochondrial gene expression were filtered. Cells with >700 detected genes and genes detected in >3 cells were reserved. The “DoubletFinder” package (14) was used to remove samples with a doublet rate >0.4%. After cell filtering, the scRNA-seq data of high-quality cells were normalized to find highly variable genes for downstream analyses. Then, PCA was done on highly variable genes to identify significant principal components (PCs). Cell clustering was undertaken on the top-20 PCs using the t-SNE algorithm. The “FindAllMarkers” function was applied to detect the marker genes of each cell cluster. Next, annotation of cell type in different cell clusters was done with the “SingleR” package (15). HCC-related clinical information and gene-expression data were downloaded from The Cancer Genome Atlas (TCGA) database (www.cancer.gov/), GEO database (GSE76427), and the International Cancer Genome Consortium (ICGC) database (https://dcc.icgc.org/), and only HCC samples with complete survival information were retained. Then, the TCGA-LIHC dataset (which contains the survival data and clinical information for 368 HCC patients) was used as the training set. Gene-expression data from TCGA-LIHC were downloaded in the format of fragments per kilobase million and analyzed. Data on progression-free survival (PFS), disease-specific survival (DSS), and disease-free survival (DFS) for TCGA-LIHC were downloaded from UCSC Xena (https://xena.ucsc.edu/) (16). The ICGC-LIRI-JP dataset and GES76427 dataset contain the survival data, clinical information, and gene-expression data for 232 and 115 HCC patients, respectively. The mRNA-seq data in the ICGC-LIRI-JP dataset were transformed by log_2_(x+1), and data in the GSE76427 dataset were normalized using the “limma” package (17). Then, the batch effect between the ICGC-LIRI-JP dataset and GSE76427 dataset was eliminated using the “sva” package (https://bioconductor.org/packages/sva/), so that they were combined into a merged dataset to serve as the test set. A summary of the clinicopathological characteristics of patients in all datasets is shown in **Supplementary Table S1**.

### Macrophage infiltration and related survival analyses

The relative content of M1 and M2 macrophages in each TCGA-LIHC sample was calculated on CIBERSORTx (https://cibersortx.stanford.edu/) (18) using the default signature matrix. The “surv_cutpoint” function of the “survminer” package (https://rdocumentation.org/packages/survminer/) was used to calculate the optimal cutoff value to distinguish high- and low-content groups of M1 or M2 macrophages in TCGA-LIHC samples. Survival analyses were carried out using the “survival” package (https://cran.r-project.org/web/packages/survival/index.html). Survival between low- and high-M1 (or M2) macrophage-content groups were analyzed and compared by Kaplan–Meier method to ascertain if M1 and/or M2 macrophage content was related to survival from HCC.

### Acquisition of M2-like TAM-related genes

After grouping the HCC samples by trait of high or low M2 macrophage content, we analyzed TCGA-LIHC expression data using the Weighted Gene Co-expression Network Analysis (“WGCNA”) package (19) to obtain genes most related to M2 macrophage content. Samples were clustered to ascertain the overall relevance of all samples in the dataset, and outliers were excluded. The soft thresholding power β was chosen based on the lowest power for which the scale-free topology fit index reached a high value. The minimum gene number/module was set to 50 and, finally, 11 modules were generated. Next, we undertook correlation analyses between modules and traits to find the most relevant modules for M2 macrophage content. Finally, the obtained modular genes were intersected with the TAM marker genes acquired from analyses of scRNA-seq data to filter M2-like TAM-related genes.

### Construction and validation of a M2-like TAM-related prognostic signature

To obtain M2-like TAM-related genes that could construct a prognostic signature, univariate Cox regression and least absolute shrinkage and selection operator (LASSO) regression analyses were carried out. Initially, we wished to uncover the association between signature genes and the prognosis. Hence, after the consensus clustering of HCC samples into different clusters based on expression of signature genes, we analyzed the difference in the prognosis among clusters. And we undertook analyses of the enrichment of function and signaling pathways of signature genes using the Gene Ontology (GO) database (http://geneontology.org/) and Kyoto Encyclopedia of Genes and Genomes (KEGG) database (www.genome.jp/kegg/), respectively, by employing the “clusterProfiler” package (20). To group the HCC patients, the risk score of each HCC patient in the training set was calculated according to the following formula:


$$\text{risk score}=\sum_{i=1}^{n}\;\left[coefficient\;\left(genei\right)*expression\;\left(genei\right)\right]$$

Then, patients were divided into low- and high-risk groups based on the optimal cutoff of the risk score. Kaplan–Meier survival curves and the log-rank test were used to analyze and compare the survival between low- and high-risk groups. Receiver operator characteristic (ROC) curves for 1, 3, and 5 years were plotted using the “survivalROC” package (https://cran.rstudio.com/web/packages/survivalROC/index.html/) to evaluate the performance of the prognostic signature. According to the KEGG database, signaling pathways enriched significantly in low- and high-risk groups were analyzed by gene set enrichment analysis (GSEA) software (21). The count of permutations was set to 1000. Significantly enriched pathways were defined as those with P<0.05 and a false discovery rate<0.25. The five most enriched pathways in the high- and low-risk groups, respectively, were obtained. Moreover, the prognostic signature was validated in the test set. Based on the prognostic signature and clinical characteristics of samples, the “rms” package (https://cran.r-project.org/web/packages/rms/index.html) was used to construct a nomogram. The performance of the nomogram was evaluated using calibration curves and 1-, 3-, and 5-year ROC curves.

### Analyses of immune cells, immune functions, and immunotherapy

The “GSVA” (22) and “GSEABase” packages (www.bioconductor.org/packages/release/bioc/html/GSEABase.html/) were used to analyze differences in scores for immune cells and immune function between high- and low-risk groups. The Tumor Immune Dysfunction and Exclusion (TIDE) score was calculated for each sample in high- and low-risk groups on the TIDE website (http://tide.dfci.harvard.edu/) (23). The immunophenoscore of each sample was obtained on The Cancer Immunome Atlas (TCIA) database (https://tcia.at/home/) (24).

### Correlation analyses and drug screening

We wished to further identify new potential targets and more efficacious drugs for HCC treatment. The CellMiner database (https://discover.nci.nih.gov/cellminer/) was employed to screen for antitumor drugs whose sensitivity was associated significantly with prognostic genes. The “pRRophitic” package (https://github.com/paulgeeleher/pRRophetic/) was used to predict the half-maximal inhibitory concentration (IC_50_) of different drugs in high- and low-risk groups. The lower the IC_50_ of a drug, the more efficacious the drug is for treating cancer.

### Sample collection and real-time reverse transcription-quantitative polymerase chain reaction

The study protocol was approved by the Human Subjects Committee of Xijing Hospital (Xian, China). All patients provided written informed consent. We collected samples of tumor tissue and adjacent normal tissue from 15 patients with HCC. Detailed clinicopathological information is summarized in **Supplementary Table S2**. Total RNA from human tissues was extracted using TRIzol^®^ Reagent (Invitrogen). Then, the RNA was reverse-transcribed into complimentary-DNA using a PrimeScript RT kit (Takara Biotechnology. Shiga, Japan). qPCR was done using SYBR Premix Ex Taq II (Takara Biotechnology) for a real-time PCR detection system (Bio-Rad Laboratories, Hercules, CA, USA). **Supplementary Table S3** lists all the primers used in PCR. Expression of genes was normalized to that of glyceraldehyde 3-phosphate dehydrogenase (GAPDH).

### Statistical analyses

Statistical analyses were conducted using R 4.0.3 (R Institute for Statistical Computing, Vienna, Austria). The packages within R used for statistical analyses were as described above. The Kaplan–Meier method was employed for survival analyses. The Wilcoxon test was used to compare differences between two groups. The Kruskal–Wallis test was employed to compare differences among three or more groups. P<0.05 was considered significant.

## Results

### Screening for M2 macrophage-related genes by WGCNA in HCC

**Figure 2:**
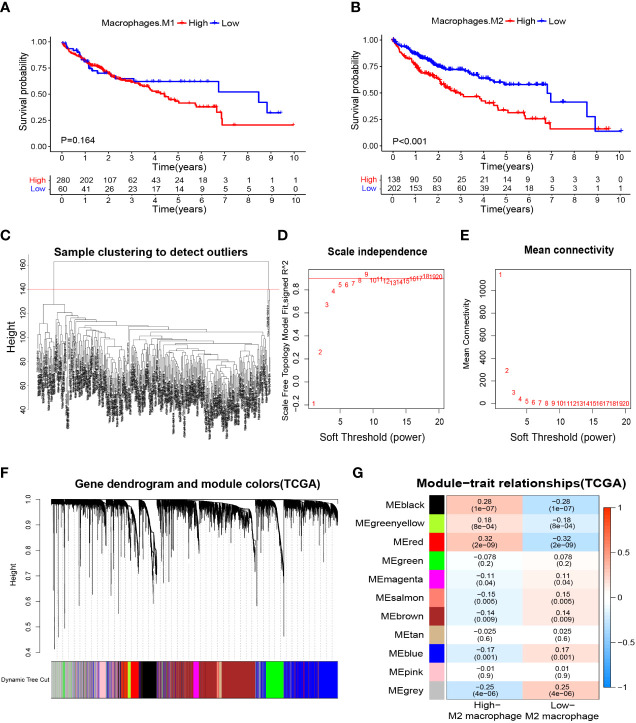
Macrophage-related survival analysis and screening of M2 macrophage related genes. **(A)** Kaplan–Meier survival curves showed no difference in the prognosis between groups with high and low content of M1 macrophages. **(B)** The prognosis was significantly worse in the group with high content of M2 macrophages. **(C)** Samples were clustered and outlier samples were not found. **(D, E)** According to the instructions of the WGCNA package, 7 was selected as the soft threshold power. **(F, G)** Correlation analysis of modules with traits yielded 10 non-gray modules, with the red module considered to be the most relevant module for M2 macrophages. WGCNA, weighted gene co-expression network analysis.

We wished to further clarify the relationship between macrophages and the HCC prognosis. The CIBERSORTx algorithm was used to calculate the content of M1 and M2 macrophages in TCGA-LIHC samples. Then, HCC patients were divided into high- and low-M1 macrophage-content groups and high- and low-M2 macrophage-content groups. Kaplan–Meier analyses showed no significant difference in survival from HCC between high- and low-M1 macrophage-content groups (**Figure 2A**), but HCC patients in the low-M2 macrophage-content group had longer survival (**Figure 2B**), thereby indicating that M2 macrophages had an important role in HCC. Based on this observation, WGCNA was undertaken to identify M2 macrophage-related genes in HCC. First, no outlier was detected in TCGA-HCC (**Figure 2C**), and 7 was chosen as the optimal soft-threshold power (**Figures 2D, E**), and 11 modules were identified by WGCNA (**Figures 2F, G**). Correlation analysis between modules and M2 macrophage content showed the red module to be associated most significantly with high-content M2 macrophages (correlation = 0.32, P<0.001). Thus, 405 genes (**Supplementary Table S4**) in the red module were selected for downstream analyses.

### Acquisition of TAM marker genes using scRNA-seq data

**Figure 3:**
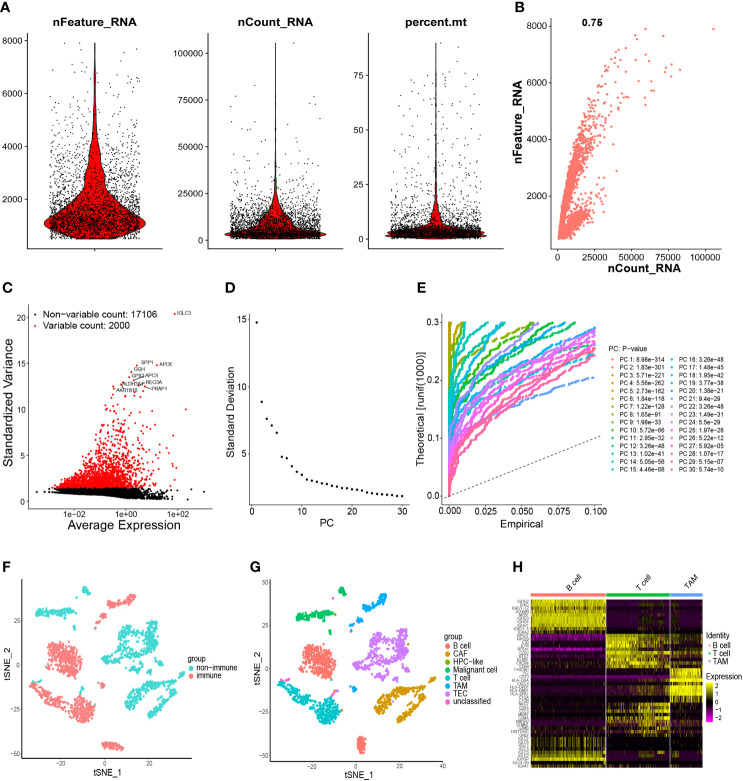
Processing of scRNA-seq data and acquisition of TAM marker genes. **(A)** Quality control of scRNA-seq data of samples of HCC cells. **(B)** The number of genes detected was positively associated with the depth of sequencing. **(C)** Scatter plots showing the top-2000 differentially expressed genes. **(D, E)** Principal component analysis was employed to classify the cells, and the top-30 PCs are displayed. **(F)** Initially, cells were annotated as “immune cells” and “non-immune cells” by the t-SNE algorithm. **(G)** Further detailed annotation of cells. **(H)** Heatmap demonstrated the marker genes with differential expression in immune cells. scRNA-seq, single-cell RNA-seq; TAM, tumor-associated macrophage; HCC, hepatocellular carcinoma; PCs, principal components; t-SNE, t-distributed stochastic neighbor embedding.

After quality control, 19,106 genes within 2,719 cells were obtained. The number of genes (nFeature), the sequence count per cell (nCount), and percentage of mitochondrial genes (percent.mt) were displayed in Vlnplots (**Figure 3A**). Correlation analyses showed that nCount was correlated positively with nFeature (**Figure 3B**). Then, 2000 variable genes were plotted in a scatter diagram (**Figure 3C**). Thirty PCs were identified (**Figures 3D, E**), showing high heterogeneity in HCC cells. The top-20 PCs were selected for t-SNE analyses. According to t-SNE and cell-type annotation, HCC cells were clustered into two groups: 1,226 immune cells and 1,493 non-immune cells (**Figure 3F**). The immune group was composed of B cells, T cells, and TAMs. The non-immune group included cancer-associated fibroblasts (CAFs), cells with an unknown entity but express hepatic progenitor cell markers (HPC-like cells), malignant cells, tumor-associated endothelial cells (TECs), and unclassified cells (**Figure 3G**). The 2047 TAM marker genes of immune cells were detected (**Supplementary Table S5**) and shown in a heatmap (**Figure 3H**).

### Screening of M2-like TAM-related prognostic genes

**Figure 4:**
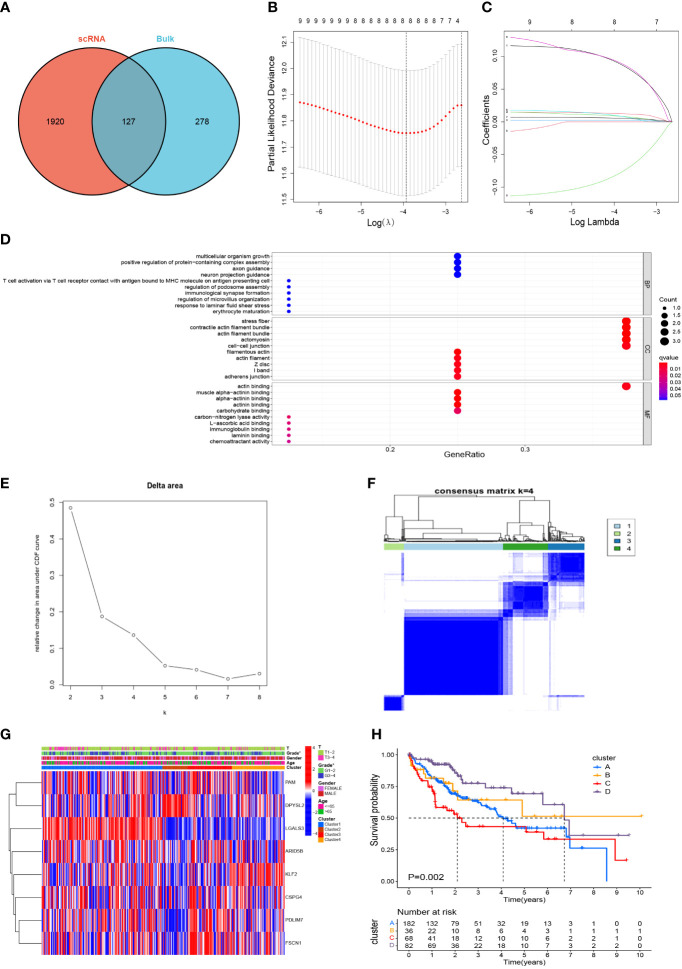
Screening of M2-like TAM-related prognostic genes and unsupervised consensus clustering. **(A)** Acquisition of candidate M2-like TAM-related genes. **(B, C)** lasso regression analysis to identify signature genes. **(D)** Enrichment analysis using the GO database. **(E, F)** Consensus clustering plot showing that 4 was the optimal k value and TCGA-LIHC samples were classified into four clusters. **(G)** Heatmap demonstrated the differences in gene expression and clinicopathological characteristics among the four clusters. **(H)** Kaplan–Meier survival curves revealed survival differences between the four clusters. *P<0.05. TAM, tumor-associated macrophage; lasso, least absolute shrinkage and selection operator; GO, gene oncology.

After marking the intersection of 2047 TAM marker genes and 405 M2 macrophage modular genes, 127 candidate M2-like TAM-related genes were obtained (**Figure 4A**, **Supplementary Table S6**). Initially, the univariate Cox regression analysis revealed nine genes associated with the HCC prognosis (**Supplementary Table S7**). Finally, LASSO regression analysis identified eight prognostic signature genes: PDZ and LIM domain 3 (PDLIM3), peptidylglycine alpha-amidating monooxygenase (PAM), PDZ and LIM domain 7 (PDLIM7), fascin actin-bundling protein 1 (FSCN1), dihydropyrimidinase like 2 (DPYSL2), AT-rich interaction domain 5B (ARID5B), galectin 3 (LGALS3), and Kruppel-like factor 2 (KLF2) (**Figures 4B, C**). The prognostic genes were enriched significantly in 403 terms according to the GO database, the top-10 of which were displayed in a bubble plot (**Figure 4D**). The biological process (BP) category included “multicellular organism growth” and “T cell activation *via* T cell receptor contact with antigen bound to MHC molecule on antigen presenting cell”. The cell component (CC) category included “stress fiber” and “contractile actin filament bundle”. The molecular function (MF) category included “muscle alpha-actinin binding” and “alpha-actinin binding”. Moreover, TCGA-LIHC samples were consistently clustered into different clusters according to the expression of prognostic genes. It can be seen that the area under the cumulative density function (CDF) curve increased significantly when k ≤ 4 (**Figure 4E**), but the area under the CDF curve did not increase significantly when k≥5. And when k=5, the effect of consensus clustering was not good (**Supplementary Figure S2**). Therefore, HCC patients were divided into 4 clusters (**Figure 4F**). Differences in gene expression and clinicopathological characteristics among these four clusters were demonstrated with a heatmap (**Figure 4G**). Most importantly, there was a significant difference in survival between the four clusters (P=0.002) (**Figure 4H**), which initially demonstrated the prognostic value of these eight genes.

### Construction of a M2-like TAM-related prognostic signature

**Figure 5:**
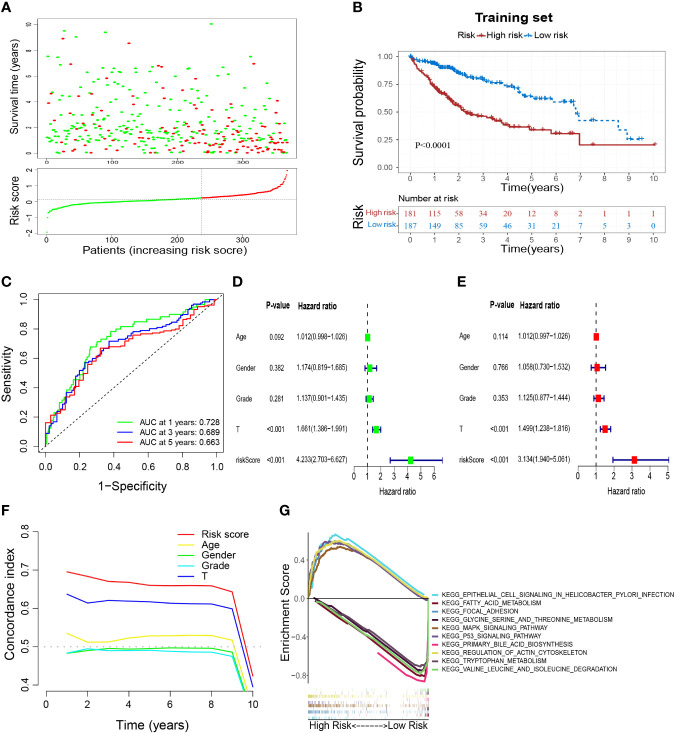
Construction of a M2-like TAM-related prognostic signature. **(A)** Survival status and risk scores of HCC patients in high- and low-risk groups in the training set. Green dots denote low risk and red dots denote high risk. **(B)** Kaplan–Meier survival curves showed a significantly worse prognosis for the high-risk group in the training set. **(C)** ROC curves for 1, 3, and 5 years and their AUCs. **(D–F)** The results of univariate analysis, multivariate analysis, and C-index indicated that risk score was an independent risk factor influencing survival status in preference to other indicators. **(G)** Results of GSEA analysis. TAM, tumor-associated macrophage; HCC, hepatocellular carcinoma; ROC, receiver operator characteristic; AUC, area under curve; C-index, Concordance index; GSEA, gene set enrichment analysis.

According to the coefficients (**Table 1**) and expression of prognostic genes, the risk score of each sample in TCGA-HCC was calculated. Then, HCC patients in the training set were divided into low- and high-risk groups based on the optimal cutoff of risk score (0.126) (**Figure 5A**). Overall survival (OS) (**Figure 5B**), DFS, PFS, and DSS (**Supplementary Figures S3A–C**) were longer in the low-risk group than in the high-risk group, which indicated that patients in the low-risk group had a better overall prognosis. To evaluate the performance of the risk model, ROC curves were plotted, and the area under the ROC curve (AUC) at 1, 3, and 5 years was 0.728, 0.689, and 0.663, respectively (**Figure 5C**). The results for univariate and multivariate analyses (**Figures 5D, E**) and the Concordance index (C-index) (**Figure 5F**) showed that the risk score was: (i) an independent factor affecting survival; (ii) a superior prognostic predictor than other indicators. Moreover, GSEA showed that “epithelial cell signaling in *Helicobacter pylori* infection”, “regulation of actin cytoskeleton”, “p53 signaling pathway”, “focal adhesion” and “MAPK signaling pathway” were enriched significantly in the high-risk group, whereas “fatty acid metabolism”, “glycine, serine and threonine metabolism”, “primary bile acid biosynthesis”, “tryptophan metabolism” and “valine, leucine and isoleucine degradation” were enriched significantly in the low-risk group (**Figure 5G**).

**Table 1:** Results of LASSO regression analysis. lasso, least absolute shrinkage and selection operator.

Gene	HR (95%CI)	P-value	Coefficient
PDLIM3	1.137 (1.019–1.270)	0.022	0.091027
PAM	1.065 (1.011–1.121)	0.017	0.012799
PDLIM7	1.039 (1.009–1.069)	0.010	0.008915
FSCN1	1.007 (1.002–1.012)	0.009	0.002318
DPYSL2	1.042 (1.007–1.077)	0.017	0.009523
ARID5B	1.114 (1.024–1.212)	0.012	0.092583
LGALS3	1.006 (1.002–1.011)	0.008	0.005666
KLF2	0.951 (0.907–0.996)	0.032	−0.08078

### External validation of the M2-like TAM-related prognostic signature

**Figure 6:**
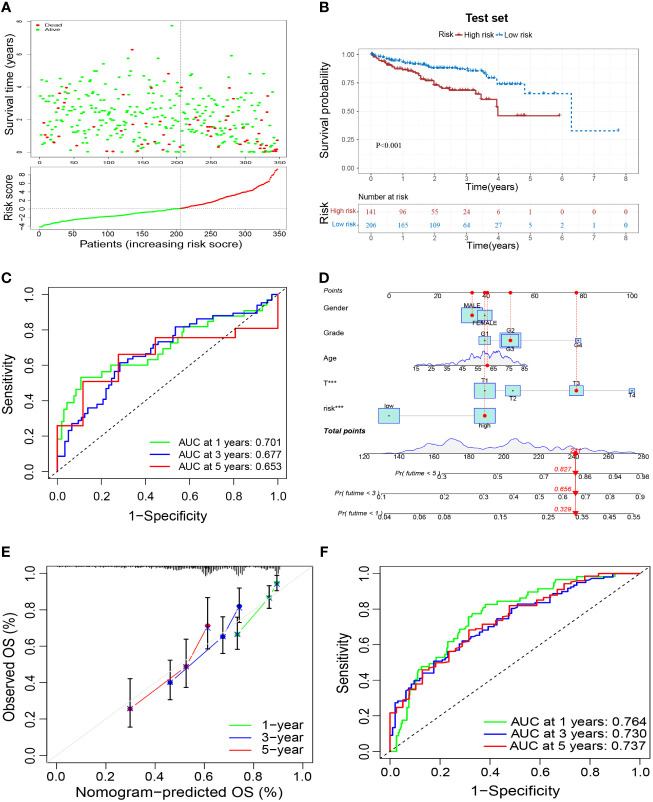
External validation of a M2-like TAM-related prognostic signature. **(A)** Survival status and risk scores of HCC patients in the high- and low-risk groups in the test set. Green dots denote low risk and red dots denote high risk. **(B)** Kaplan–Meier survival curves showed a significantly worse prognosis for the high-risk group in the test set. **(C)** ROC curves for 1, 3, and 5 years and their AUCs. **(D)** Nomogram based on risk scores and clinical indicators. The results of a calibration curve **(E)** and ROC curves **(F)** showed the reliable performance of the nomogram. TAM, tumor-associated macrophage; HCC, hepatocellular carcinoma; ROC, receiver operator characteristic; AUC, area under curve. ***P<0.001.

To verify the reliability of the prognostic signature, we further validated it in the test set. Samples were grouped in the same way as in the training set (**Figure 6A**). Patients in the high-risk group had a worse prognosis than those in the low-risk group (**Figure 6B**), and had an AUC of 0.701, 0.677, and 0.653 at 1, 3, and 5 years in the test set, respectively (**Figure 6C**). These results validated the reliability of the M2-like TAM-related prognostic signature in predicting the prognosis of HCC patients. Based on the risk score of our prognostic signature and other clinicopathological indicators of patients, we constructed a nomogram to make a more comprehensive prediction of patient survival (**Figure 6D**). Moreover, the results of the calibration curve and ROC curve of the nomogram showed a reliable performance, with an AUC of 0.764, 0.730, and 0.737 at 1, 3, and 5 years, respectively (**Figures 6E, F**).

### Analyses of clinicopathological characteristics based on the prognostic signature

**Figure 7:**
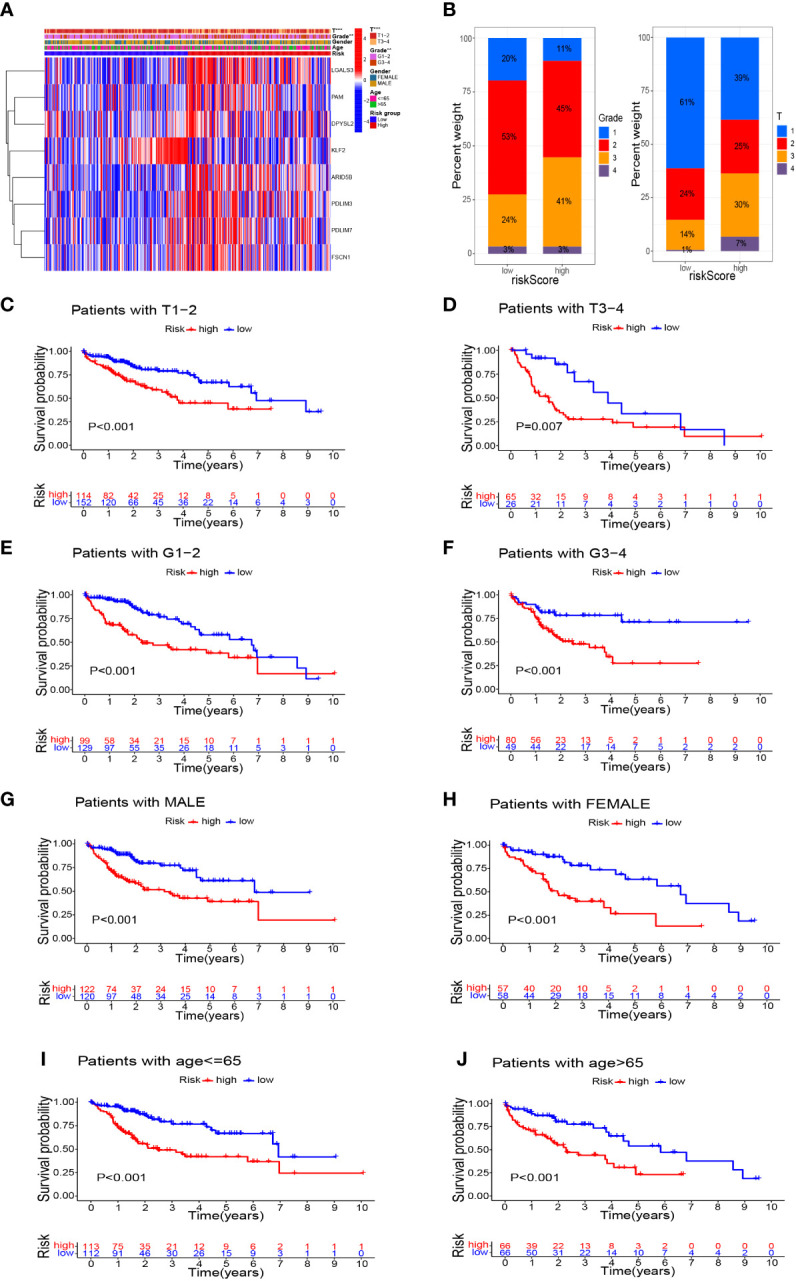
Survival analysis based on stratification of clinicopathological characteristics. **(A)** Heatmap demonstrated the differences in gene expression and clinicopathological features between high-risk and low-risk groups. **(B)** Histograms related to clinicopathological features. Kaplan–Meier survival curves illustrated the results of survival analysis stratified by T stage **(C, D)**, tumor grade **(E, F)**, sex **(G, H)**, and age **(I, J)**. **P<0.01, ***P<0.001.

In addition to significant differences in survival between high- and low-risk groups, they also differed in their clinicopathological characteristics. **Figure 7A** shows a heatmap of the clinicopathological characteristics and expression of signature-related genes in high- and low-risk groups. There were no significant differences between high- and low-risk groups in terms of sex and age distribution, whereas there were significant differences in the depth of tumor infiltration (T stage) and tumor grade, with a significantly higher proportion of patients of grade 3 and T3–4 in the high-risk group than in the low-risk group (**Figure 7B**). Survival analyses of HCC patients divided into different subgroups according to their clinicopathological indicators showed that the survival outcome of patients in the high-risk group was worse than that of the low-risk group, whether grouped by sex, age, grade, or T stage (**Figures 7C–J**).

### Risk signature-related immune cells, immune function, and the immunotherapeutic landscape

**Figure 8:**
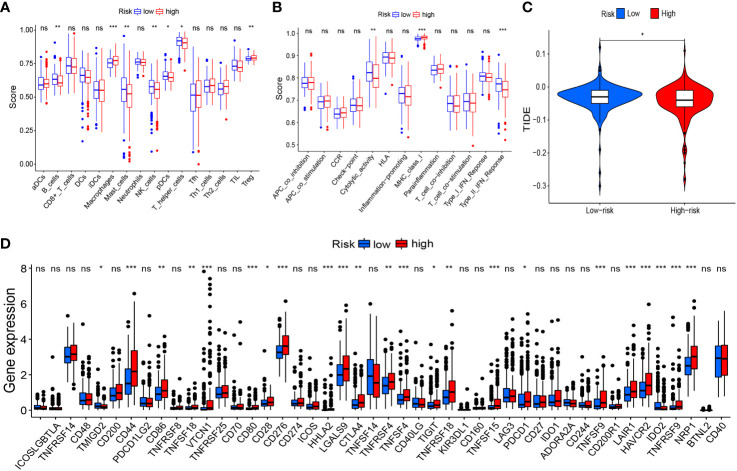
Risk signature-related immune landscapes. **(A, B)** Differences in scores of immune cells and immune function between high- and low-risk groups. **(C)** TIDE scores of high- and low-risk groups. **(D)** Differential expression of immune-checkpoint genes between high- and low-risk groups. *P<0.05, **P<0.01, ***P<0.001, ns, not significant. TIDE, Tumor Immune Dysfunction and Exclusion.

Based on risk grouping, in terms of immune cells, we discovered that the content of macrophages and T_regs_ was higher in the high-risk group than that in the low-risk group, whereas the content of B cells, mast cells, NK cells, plasmacytoid dendritic cells (pDCs), and helper T cells was lower than that in the low-risk group (**Figure 8A**). In terms of immune functions, the high-risk group was lower than the low-risk group in terms of cytolytic activity and a type-II interferon response, but higher than the low-risk group for major histocompatibility class (MHC) class-I (**Figure 8B**). With regard to immunotherapy, TIDE scores were higher in the low-risk group than those in the high-risk group (**Figure 8C**), suggesting that patients in the low-risk group were more likely to experience immune evasion, and that immunotherapy may be less efficacious. There was no significant difference in the scoring of several immunotherapy treatments between patients in high- and low-risk groups (**Supplementary Figures 1A–D**). In terms of expression of the genes associated with immune checkpoints, many differentially expressed genes between the two groups were documented (**Figure 8D**), such as CD44, CD86, and CD276, which showed significantly higher expression in the high-risk group. This finding offers the possibility of discovering new targets for immunotherapy.

### Prediction of potential anti-cancer drugs

**Figure 9:**
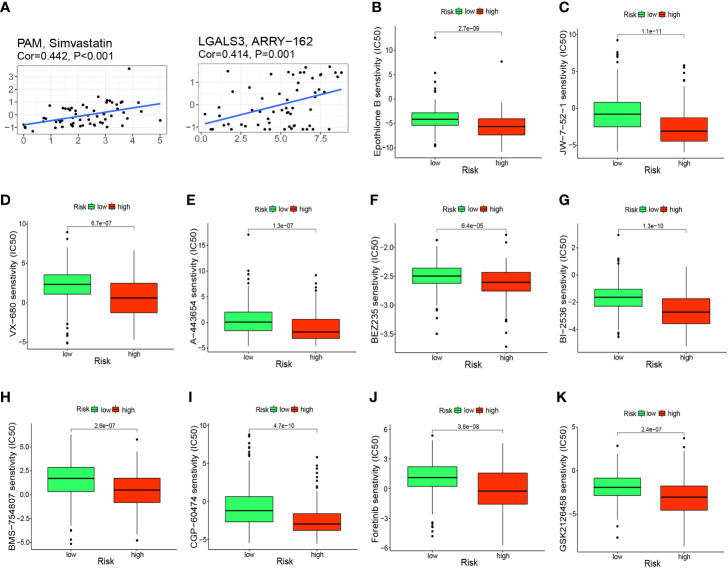
Prediction of potential anticancer drugs based on signature genes and risk groups. **(A)** PAM and LGALS3 were positively correlated with the sensitivity of simvastatin and ARRY-162, respectively. **(B–K)** 10 drugs with higher sensitivity in the high-risk group compared with the low-risk group.

To further investigate the clinical use of prognostic genes, we employed the CellMiner database to explore the relationship between prognostic genes and drug sensitivity. PAM was correlated significantly and positively with simvastatin sensitivity (correlation = 0.442, P<0.001) and LGALS3 was correlated significantly and positively with ARRY-162 sensitivity (correlation = 0.414, P<0.001) (**Figure 9A**). Patients in the high-risk group had a significantly worse prognosis, so we predicted 10 drugs with higher sensitivity in the high-risk group: epothilone B, A-443654, BEZ235, BI-2536, BMS-75480, CGP-6047, foretinib, GSK212645, JW-7-52-1, and VX-680. Epothilone B had the lowest IC_50_ (**Figures 9B–K**).

### Measurement of signature-genes expression in tissues

**Figure 10:**
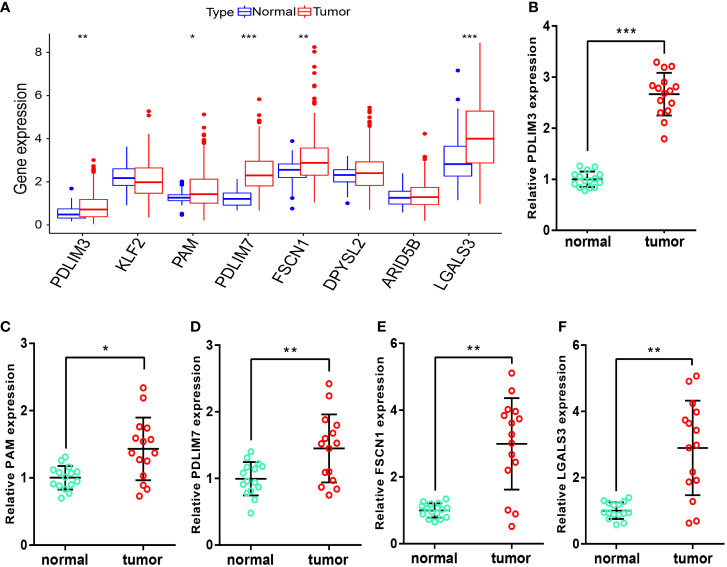
Measurement of signature-gene expression in tissues. **(A)** Expression of signature genes in TCGA-LIHC samples. **(B–F)** RNA expression of PDLIM3 **(B)**, PAM **(C)**, PDLIM7 **(D)**, FSCN1 **(E)**, and LGALS3 **(F)** in tissues. *P<0.05, **P<0.01, ***P<0.001.

After obtaining the M2-like TAM-related biomarkers and constructing related prognostic signature, we further analyzed the expression of signature genes in TCGA-LIHC samples. **Figure 10A** showed that the RNA expression levels of PDLIM3, PAM, PDLIM7, FSCN1, and LGALS3 in tumor samples were significantly upregulated. Moreover, in the samples we obtained from HCC patients, the RNA expression levels of these 5 genes (**Figures 10B–F**) were also significantly higher in tumor tissues than in adjacent normal tissues, perhaps suggesting that these genes play a role in the progression of HCC.

## Discussion

As the main type of liver cancer, HCC is thought to be related mainly to injury and long-term inflammation (25), accompanied by infiltration of various types of immune cells into liver tissue (26). The TME comprises tumor cells and non-immune cells. The interaction of tumor cells with the TME promotes HCC progression through multiple mechanisms. For example, TECs have greater proliferative capacity (27), angiogenic capacity, and drug resistance compared with those of normal endothelial cells (28). CAFs can secrete CLCF1 to regulate HCC “stemness” (29), and can also promote HCC progression by secreting proinflammatory factors such as interleukin (IL)-6 (30). TAMs (or M2 macrophages) are important components of the TME. They play an important part in HCC development, such as producing CXCL8 and IL-6 (31, 32), which enhance the invasion and metastasis of HCC cells and promote HCC progression. Moreover, TAMs have been shown to promote the angiogenic process of HCC by producing vascular endothelial growth factors (33), enhancing cell stemness by upregulating secretion of the protein S100A9 (34, 35), and even increasing drug resistance by inducing immunosuppression (36).

Due to the important role of TAMs in HCC development, there is growing interest in TAMs-based therapeutic approaches. Wang et al. found that targeted delivery of microRNA (miR)-99b to TAMs in HCC could inhibit tumor growth by inducing the conversion of macrophages from the M2 phenotype to the M1 phenotype (37). Yang et al. found that injection of compound kushen attenuated TAMs-mediated immunosuppression and increased the sensitivity of HCC to sorafenib (38). With regard to the relationship between TAMs and cancer prognosis, Hwang et al. found that a high number of M2 macrophages was associated with a worse prognosis in non-small-cell lung cancer (39). Related studies in HCC are lacking, so more in-depth studies on the relationship between TAMs and HCC prognosis are needed urgently.

We found that the prognosis of TCGA-LIHC samples with high content of M2 macrophages was significantly worse compared with samples with low content of M2 macrophages, which demonstrated the association between M2 macrophages and prognosis in HCC. Then, 127 M2-like TAM-related genes were obtained by intersecting the M2 macrophage modular genes screened from TCGA-LIHC with TAM marker genes screened from the GEO database. After univariate regression and LASSO regression analyses, eight prognosis-related genes (PDLIM3, PAM, PDLIM7, FSCN1, DPYSL2, ARID5B, LGALS3, and KLF2) were screened for construction of a prognostic signature. Among these genes, some have been reported to play an important part in HCC, but some have not been studied deeply. For example, Pu et al. found that FSCN1 restricted HCC progression after receiving upstream inhibition (40). Liu et al. found that FSCN1 overexpression promoted the migration and invasion of HCC cells (41). Bhat et al. revealed that upregulation of LGALS3 expression was associated significantly with HCC recurrence (42). Zhang et al. identified LGALS3 as a key gene in the development of bone metastases and associated skeletal complications in HCC (43). Furthermore, among our screened prognostic signature genes, KLF2 (the only protective factor for the prognosis) has been shown to inhibit the growth, migration, and metastasis of HCC cells, and its expression to be downregulated significantly in HCC (44, 45).

After unsupervised consensus clustering of TCGA-LIHC samples into four clusters based on expression of eight prognosis-related genes, Kaplan–Meier survival analyses showed significant differences between the four clusters, which suggested an association between these eight genes and the prognosis. Then, the risk scores of patients were calculated according to our prognostic signature. Patients were divided into high- and low-risk groups according to the best cutoff values. We found that patients in the high-risk group had a significantly worse prognosis than those in the low-risk group. GSEA showed that the high-risk group was more enriched in cancer-related pathways, such as the p53 pathway and mitogen-activated protein kinase pathway, whereas the low-risk group was more enriched in metabolism-related pathways, which explained (at least in part) the worse prognosis of the high-risk group. The results of the C-index, univariate analyses, multivariate analyses, and ROC curves showed that our signature could predict the prognosis of HCC independently of other indicators in the training set and had a promising performance. Moreover, we externally validated the prognostic signature in the test set consisting of the GSE76427 dataset and results from the ICGC database: their general applicability and validity were demonstrated. Based on our signature-related risk scores and clinicopathological indicators of patients, we constructed a nomogram to provide a measure by which the prognosis of the patients could be evaluated from multiple aspects.

In addition to predicting the prognosis of HCC patients effectively, our prognostic signature revealed associations of risk grouping with the immune landscape and the response to immunotherapy. In terms of immune cells, the content of B cells, mast cells, NK cells, and pDCs cells was lower in the high-risk group, whereas the content of T_regs_ was higher. With regard to immune function, MHC class I was more active in the high-risk group, whereas cytolytic activity and the type-II interferon response were more predominant in the low-risk group, which may have been related to the higher NK-cell content in the low-risk group. The relationship between immune cells in the TME and prognosis has been studied intensively in various cancer types: an increased percentage of NK cells in tumor tissue or peripheral blood may suggest a better prognosis (46, 47). In renal cancer and muscle-infiltrating bladder cancer, infiltration of mast cells is an unfavorable prognostic factor (48, 49), whereas the role in breast cancer is controversial (50). Kim et al. found that B-cell deficiency promoted the growth of head and neck squamous cell carcinoma (51), and that B cells were associated positively with a good prognosis in cancers (52), such as lung cancer (53), gastric cancer (54), and HCC (55). The pDCs infiltration in a study by Jensen et al. suggested a poor prognosis for stage-I/II melanoma (56). Conversely, Kießler and colleagues found that the degree of pDCs infiltration was correlated positively with progression-free survival and overall survival in patients with colon cancer (57). A meta-analysis of 17 cancer types by Shang et al. revealed a significant negative effect of T_regs_ on overall survival (58). Thus, the differences in the immune landscape revealed by risk grouping based on our model indicated that differences in the HCC prognosis may arise from TME heterogeneity, thereby providing new ideas for our future studies.

During screening of signature genes and undertaking risk grouping, we also analyzed and screened for potential anti-cancer drugs. We found that the sensitivity of simvastatin and ARRY-162 (i.e., binimetinib) was correlated positively with expression of PAM and LGALS3, respectively. Simvastatin has been reported to increase the sensitivity of HCC cells to sorafenib (59), induce cell-cycle arrest (60), and inhibit the growth and invasion of HCC cells (61). Binimetinib is used widely as an inhibitor of mitogen-activated protein kinase in melanoma treatment (62). A combination of binimetinib and capecitabine can enhance the anticancer effect in patients with cholangiocarcinoma (63). Therefore, our data provide further support for use of these two drugs in clinical treatment of HCC. Also, the relationship between these two drugs and signature genes merits further exploration. In our prognostic model, once a patient is classified in the high-risk group, it often denotes a worse prognosis, so screening for drugs that are more sensitive in the high-risk group may rescue their poor prognosis. Therefore, 10 drugs with higher sensitivity in the high-risk group were screened, with epothilone B (i.e., patupilone) showed significantly higher sensitivity in the high-risk group and had the lowest IC_50_ among the 10 drugs screened. Zhou et al. also found that epothilone B could inhibit the growth of HCC cells (64). After screening for M2-like TAM-related biomarkers and constructing a prognostic signature, the expression of these genes in HCC tissues remained unknown. We therefore analyzed their expression in TCGA samples and performed further validation in the tissues we collected. We found that the RNA expression levels of PDLIM3, PAM, PDLIM7, FSCN1 and LGALS3 were significantly upregulated in tumor samples, which provides ideas for further studies.

In general, the high mortality rate and poor prognosis of HCC in cancer impose a heavy burden on families and public-health systems. In recent years, increasing numbers of researchers have constructed different types of prognostic signatures for HCC patients. Tang et al. (65) and Zhang et al. (66) focused on hepatitis C virus-associated HCC (HCV-HCC). They identified hub genes that play a key part in HCV-HCC and constructed related prognostic models. Tang et al. (67) screened the relevant genes from the perspective of the immunological phenotype of tumors to construct prognostic models and predict immunotherapy effects and drug candidates. Li et al. (68) revealed the prognostic differences among different phenotypes of CpG-island methylation in HCC patients, and screened the associated genes to construct a prognostic signature. Dai et al. (69) and Rao et al. (70) screened prognostic-related genes from metabolic- and aerobic respiration-related perspectives, respectively, to construct models. Those studies refine prediction of the prognosis of HCC patients from various perspectives and their models have good efficacy. Similar to our study (at least in part), they used the results of bulk-seq from public databases in the construction of their prognostic signature. Bulk-seq gives the total expression of genes in tissues, but the transcriptome of different cell types and proportions within tissues are not revealed. Therefore, different from the literature, we integrated single-cell sequencing (which enables identification of cell types and gives the expression profile at cellular resolution) with bulk-seq to identify specific M2-like TAM prognostic biomarkers for HCC. To our knowledge, this was the first study to use scRNA-seq and bulk-seq data to: (i) screen for M2-like TAM-related genes; (ii) construct a prognostic model in HCC. These signature genes facilitate deeper understanding and investigation of HCC. The prognostic signature we identified could aid the clinical management of HCC.

The construction and external validation of our prognostic model were based on data from TCGA, GEO and ICGC databases. However, the results from these databases are retrospective and the stability of signature performance must be confirmed in a prospective study.

## Conclusions

We constructed an M2-like TAM-associated prognostic signature. This could be a promising tool for predicting the prognosis of patients with HCC. This prognostic signature also reveals the TME to some extent, and provides potential targets for HCC treatment.

## Data availability statement

The datasets presented in this study can be found in online repositories. The names of the repository/repositories and accession number(s) can be found in the article/**Supplementary Material**.

## Ethics statement

The studies involving human participants were reviewed and approved by Human Subjects Committee of Xijing Hospital. The patients/participants provided their written informed consent to participate in this study.

## Author contributions

XQ, XZ, KL, and YS designed the study. XQ, XZ, KL, NW, XL, SL, and LZ collected and analyzed the data. XQ wrote the manuscript. YS revised and edited the manuscript. All authors contributed to the article and approved the submitted version.

## Funding

This work was supported by grants from National Natural Science Foundation of China (8217031045 and 81873554 to YQS).

## Conflict of interest

The authors declare that the research was conducted in the absence of any commercial or financial relationships that could be construed as a potential conflict of interest.

## Publisher’s note

All claims expressed in this article are solely those of the authors and do not necessarily represent those of their affiliated organizations, or those of the publisher, the editors and the reviewers. Any product that may be evaluated in this article, or claim that may be made by its manufacturer, is not guaranteed or endorsed by the publisher.

## Supplementary material

Supplementary Figure 1
**(A–D)** Scores for immunotherapy from the TCIA database. TCIA, The Cancer Immunome Atlas.

Supplementary Figure 2
**(A–H)** The results of consensus clustering.

Supplementary Figure 3 Kaplan–Meier survival curves of risk score for DFS **(A)**, PFS **(B)**, and DSS **(C)** in TCGA-LIHC cohort. DFS, disease-free survival; PFS, progression-free survival; DSS, disease-specific survival.

Supplementary Table 1 Demographic information of hepatocellular carcinoma patients in this study.

Supplementary Table 2 Clinicopathological information of 15 patients with hepatocellular carcinoma in this study.

Supplementary Table 3 The primers sequences used in this study.

Supplementary Table 4 List of 405 genes from the red module of the WGCNA analysis. WGCNA, weighted gene co-expression network analysis.

Supplementary Table 5 List of 2047 TAM marker genes obtained from scRNA-seq data analysis

Supplementary Table 6 List of 127 intersecting genes.

Supplementary Table 7 Results of univariate Cox regression analysis.

## References

[1] Sung H Ferlay J Siegel RL Laversanne M Soerjomataram I Jemal A. Global cancer statistics 2020: Globocan estimates of incidence and mortality worldwide for 36 cancers in 185 countries. CA: Cancer J Clin (2021) 71(3):209–49. doi: 10.3322/caac.21660 33538338

[2] Yang JD Hainaut P Gores GJ Amadou A Plymoth A Roberts LR. A global view of hepatocellular carcinoma: Trends, risk, prevention and management. Nat Rev Gastroenterol Hepatol (2019) 16(10):589–604. doi: 10.1038/s41575-019-0186-y 31439937 PMC6813818

[3] Altekruse SF McGlynn KA Reichman ME. Hepatocellular carcinoma incidence, mortality, and survival trends in the united states from 1975 to 2005. J Clin Oncol: Off J Am Soc Clin Oncol (2009) 27(9):1485–91. doi: 10.1200/jco.2008.20.7753 PMC2668555 19224838

[4] Tabrizian P Jibara G Shrager B Schwartz M Roayaie S. Recurrence of hepatocellular cancer after resection: Patterns, treatments, and prognosis. Ann Surg (2015) 261(5):947–55. doi: 10.1097/sla.0000000000000710 25010665

[5] Bruni D Angell HK Galon J. The immune contexture and immunoscore in cancer prognosis and therapeutic efficacy. Nat Rev Cancer (2020) 20(11):662–80. doi: 10.1038/s41568-020-0285-7 32753728

[6] Chávez-Galán L Olleros ML Vesin D Garcia I. Much more than M1 and M2 macrophages, there are also Cd169(+) and tcr(+) macrophages. Front Immunol (2015) 6:263. doi: 10.3389/fimmu.2015.00263 26074923 PMC4443739

[7] Kessenbrock K Plaks V Werb Z. Matrix metalloproteinases: Regulators of the tumor microenvironment. Cell (2010) 141(1):52–67. doi: 10.1016/j.cell.2010.03.015 20371345 PMC2862057

[8] Cassetta L Pollard JW. Targeting macrophages: Therapeutic approaches in cancer. Nat Rev Drug Discovery (2018) 17(12):887–904. doi: 10.1038/nrd.2018.169 30361552

[9] Han L Wang S Wei C Fang Y Huang S Yin T. Tumour microenvironment: A non-negligible driver for epithelial-mesenchymal transition in colorectal cancer. Expert Rev Mol Med (2021) 23:e16. doi: 10.1017/erm.2021.13 34758892

[10] Petty AJ Yang Y. Tumor-associated macrophages: Implications in cancer immunotherapy. Immunotherapy (2017) 9(3):289–302. doi: 10.2217/imt-2016-0135 28231720 PMC5619052

[11] Chen G Ning B Shi T. Single-cell rna-seq technologies and related computational data analysis. Front Genet (2019) 10:317. doi: 10.3389/fgene.2019.00317 31024627 PMC6460256

[12] Ma L Hernandez MO Zhao Y Mehta M Tran B Kelly M. Tumor cell biodiversity drives microenvironmental reprogramming in liver cancer. Cancer Cell (2019) 36(4):418–30.e6. doi: 10.1016/j.ccell.2019.08.007 31588021 PMC6801104

[13] Hao Y Hao S Andersen-Nissen E Mauck WM 3rd Zheng S Butler A. Integrated analysis of multimodal single-cell data. Cell (2021) 184(13):3573–87.e29. doi: 10.1016/j.cell.2021.04.048 34062119 PMC8238499

[14] McGinnis CS Murrow LM Gartner ZJ. Doubletfinder: Doublet detection in single-cell rna sequencing data using artificial nearest neighbors. Cell Syst (2019) 8(4):329–37.e4. doi: 10.1016/j.cels.2019.03.003 30954475 PMC6853612

[15] Aran D Looney AP Liu L Wu E Fong V Hsu A. Reference-based analysis of lung single-cell sequencing reveals a transitional profibrotic macrophage. Nat Immunol (2019) 20(2):163–72. doi: 10.1038/s41590-018-0276-y PMC6340744 30643263

[16] Goldman MJ Craft B Hastie M Repečka K McDade F Kamath A. Visualizing and interpreting cancer genomics data *Via* the xena platform. Nat Biotechnol (2020) 38(6):675–8. doi: 10.1038/s41587-020-0546-8 PMC7386072 32444850

[17] Ritchie ME Phipson B Wu D Hu Y Law CW Shi W. Limma powers differential expression analyses for rna-sequencing and microarray studies. Nucleic Acids Res (2015) 43(7):e47. doi: 10.1093/nar/gkv007 25605792 PMC4402510

[18] Newman AM Steen CB Liu CL Gentles AJ Chaudhuri AA Scherer F. Determining cell type abundance and expression from bulk tissues with digital cytometry. Nat Biotechnol (2019) 37(7):773–82. doi: 10.1038/s41587-019-0114-2 PMC6610714 31061481

[19] Langfelder P Horvath S. Wgcna: An r package for weighted correlation network analysis. BMC Bioinf (2008) 9:559. doi: 10.1186/1471-2105-9-559 PMC2631488 19114008

[20] Yu G Wang LG Han Y He QY. Clusterprofiler: An r package for comparing biological themes among gene clusters. Omics: J Integr Biol (2012) 16(5):284–7. doi: 10.1089/omi.2011.0118 PMC3339379 22455463

[21] Subramanian A Tamayo P Mootha VK Mukherjee S Ebert BL Gillette MA. Gene set enrichment analysis: A knowledge-based approach for interpreting genome-wide expression profiles. Proc Natl Acad Sci USA (2005) 102(43):15545–50. doi: 10.1073/pnas.0506580102 PMC1239896 16199517

[22] Hänzelmann S Castelo R Guinney J. Gsva: Gene set variation analysis for microarray and rna-seq data. BMC Bioinf (2013) 14:7. doi: 10.1186/1471-2105-14-7 PMC3618321 23323831

[23] Fu J Li K Zhang W Wan C Zhang J Jiang P. Large-Scale public data reuse to model immunotherapy response and resistance. Genome Med (2020) 12(1):21. doi: 10.1186/s13073-020-0721-z 32102694 PMC7045518

[24] Charoentong P Finotello F Angelova M Mayer C Efremova M Rieder D. Pan-cancer immunogenomic analyses reveal genotype-immunophenotype relationships and predictors of response to checkpoint blockade. Cell Rep (2017) 18(1):248–62. doi: 10.1016/j.celrep.2016.12.019 28052254

[25] Yu LX Ling Y Wang HY. Role of nonresolving inflammation in hepatocellular carcinoma development and progression. NPJ Precis Oncol (2018) 2(1):6. doi: 10.1038/s41698-018-0048-z 29872724 PMC5871907

[26] Landskron G de la Fuente M Thuwajit P Thuwajit C Hermoso MA. Chronic inflammation and cytokines in the tumor microenvironment. J Immunol Res (2014) 2014:149185. doi: 10.1155/2014/149185 24901008 PMC4036716

[27] Hida K Hida Y Amin DN Flint AF Panigrahy D Morton CC. Tumor-associated endothelial cells with cytogenetic abnormalities. Cancer Res (2004) 64(22):8249–55. doi: 10.1158/0008-5472.Can-04-1567 15548691

[28] Xiong YQ Sun HC Zhang W Zhu XD Zhuang PY Zhang JB. Human hepatocellular carcinoma tumor-derived endothelial cells manifest increased angiogenesis capability and drug resistance compared with normal endothelial cells. Clin Cancer Res: Off J Am Assoc Cancer Res (2009) 15(15):4838–46. doi: 10.1158/1078-0432.Ccr-08-2780 19638466

[29] Song M He J Pan QZ Yang J Zhao J Zhang YJ. Cancer-associated fibroblast-mediated cellular crosstalk supports hepatocellular carcinoma progression. Hepatol (Baltimore Md) (2021) 73(5):1717–35. doi: 10.1002/hep.31792 33682185

[30] Fang T Lv H Lv G Li T Wang C Han Q. Tumor-derived exosomal mir-1247-3p induces cancer-associated fibroblast activation to foster lung metastasis of liver cancer. Nat Commun (2018) 9(1):191. doi: 10.1038/s41467-017-02583-0 29335551 PMC5768693

[31] Yin Z Huang J Ma T Li D Wu Z Hou B. Macrophages activating chemokine (C-X-C motif) ligand 8/Mir-17 cluster modulate hepatocellular carcinoma cell growth and metastasis. Am J Trans Res (2017) 9(5):2403–11. PMC5446522 28559990

[32] Jiang J Wang GZ Wang Y Huang HZ Li WT Qu XD. Hypoxia-induced Hmgb1 expression of hcc promotes tumor invasiveness and metastasis *Via* regulating macrophage-derived il-6. Exp Cell Res (2018) 367(1):81–8. doi: 10.1016/j.yexcr.2018.03.025 29571949

[33] Deryugina EI Quigley JP. Tumor angiogenesis: Mmp-mediated induction of intravasation- and metastasis-sustaining neovasculature. Matrix Biol: J Int Soc Matrix Biol (2015) 44-46:94–112. doi: 10.1016/j.matbio.2015.04.004 PMC5079283 25912949

[34] Wan S Zhao E Kryczek I Vatan L Sadovskaya A Ludema G. Tumor-associated macrophages produce interleukin 6 and signal *Via* Stat3 to promote expansion of human hepatocellular carcinoma stem cells. Gastroenterology (2014) 147(6):1393–404. doi: 10.1053/j.gastro.2014.08.039 PMC4253315 25181692

[35] Wei R Zhu WW Yu GY Wang X Gao C Zhou X. S100 calcium-binding protein A9 from tumor-associated macrophage enhances cancer stem cell-like properties of hepatocellular carcinoma. Int J Cancer (2021) 148(5):1233–44. doi: 10.1002/ijc.33371 33205453

[36] Yao W Ba Q Li X Li H Zhang S Yuan Y. A natural Ccr2 antagonist relieves tumor-associated macrophage-mediated immunosuppression to produce a therapeutic effect for liver cancer. EBioMedicine (2017) 22:58–67. doi: 10.1016/j.ebiom.2017.07.014 28754304 PMC5552238

[37] Wang L Hu YY Zhao JL Huang F Liang SQ Dong L. Targeted delivery of mir-99b reprograms tumor-associated macrophage phenotype leading to tumor regression. J Immunother Cancer (2020) 8(2):e000517. doi: 10.1136/jitc-2019-000517 32948650 PMC7511616

[38] Yang Y Sun M Yao W Wang F Li X Wang W. Compound kushen injection relieves tumor-associated macrophage-mediated immunosuppression through Tnfr1 and sensitizes hepatocellular carcinoma to sorafenib. J Immunother Cancer (2020) 8(1):e000317. doi: 10.1136/jitc-2019-000317 32179631 PMC7073790

[39] Hwang I Kim JW Ylaya K Chung EJ Kitano H Perry C. Tumor-associated macrophage, angiogenesis and lymphangiogenesis markers predict prognosis of non-small cell lung cancer patients. J Trans Med (2020) 18(1):443. doi: 10.1186/s12967-020-02618-z PMC7686699 33228719

[40] Pu J Zhang Y Wang A Qin Z Zhuo C Li W. Adora2a-As1 restricts hepatocellular carcinoma progression *Via* binding hur and repressing Fscn1/Akt axis. Front Oncol (2021) 11:754835. doi: 10.3389/fonc.2021.754835 34733789 PMC8558402

[41] Liu Y Hong W Zhou C Jiang Z Wang G Wei G. Mir-539 inhibits Fscn1 expression and suppresses hepatocellular carcinoma migration and invasion. Oncol Rep (2017) 37(5):2593–602. doi: 10.3892/or.2017.5549 PMC5428223 28393215

[42] Bhat M Clotet-Freixas S Baciu C Pasini E Hammad A Ivanics T. Combined Proteomic/Transcriptomic signature of recurrence post-liver transplantation for hepatocellular carcinoma beyond Milan. Clin Proteomics (2021) 18(1):27. doi: 10.1186/s12014-021-09333-x 34794390 PMC8600773

[43] Zhang S Xu Y Xie C Ren L Wu G Yang M. Rnf219/α-Catenin/Lgals3 axis promotes hepatocellular carcinoma bone metastasis and associated skeletal complications. Adv Sci (Weinheim Baden-Wurttemberg Germany) (2021) 8(4):2001961. doi: 10.1002/advs.202001961 PMC7887580 33643786

[44] Lin J Tan H Nie Y Wu D Zheng W Lin W. Krüppel-like factor 2 inhibits hepatocarcinogenesis through negative regulation of the hedgehog pathway. Cancer Sci (2019) 110(4):1220–31. doi: 10.1111/cas.13961 PMC6447955 30719823

[45] Huang MD Chen WM Qi FZ Sun M Xu TP Ma P. Long non-coding rna Tug1 is up-regulated in hepatocellular carcinoma and promotes cell growth and apoptosis by epigenetically silencing of Klf2. Mol Cancer (2015) 14:165. doi: 10.1186/s12943-015-0431-0 26336870 PMC4558931

[46] Ishigami S Natsugoe S Tokuda K Nakajo A Xiangming C Iwashige H. Clinical impact of intratumoral natural killer cell and dendritic cell infiltration in gastric cancer. Cancer Lett (2000) 159(1):103–8. doi: 10.1016/s0304-3835(00)00542-5 10974412

[47] Xie MZ Tang YP Hu BL Li KZ Li JL Liang XQ. Percentage of natural killer (Nk) cells in peripheral blood is associated with prognosis in patients with gastric cancer: A retrospective study from a single center. Med Sci Monitor: Int Med J Exp Clin Res (2021) 27:e927464. doi: 10.12659/msm.927464 PMC7849206 33500378

[48] Cherdantseva TM Bobrov IP Avdalyan AM Klimachev VV Kazartsev AV Kryuchkova NG. Mast cells in renal cancer: Clinical morphological correlations and prognosis. Bull Exp Biol Med (2017) 163(6):801–4. doi: 10.1007/s10517-017-3907-7 29063337

[49] Liu Z Zhu Y Xu L Zhang J Xie H Fu H. Tumor stroma-infiltrating mast cells predict prognosis and adjuvant chemotherapeutic benefits in patients with muscle invasive bladder cancer. Oncoimmunology (2018) 7(9):e1474317. doi: 10.1080/2162402x.2018.1474317 30393586 PMC6209422

[50] Ribatti D Annese T Tamma R. Controversial role of mast cells in breast cancer tumor progression and angiogenesis. Clin Breast Cancer (2021) 21(6):486–91. doi: 10.1016/j.clbc.2021.08.010 34580034

[51] Kim SS Shen S Miyauchi S Sanders PD Franiak-Pietryga I Mell L. B cells improve overall survival in hpv-associated squamous cell carcinomas and are activated by radiation and pd-1 blockade. Clin Cancer Res: Off J Am Assoc Cancer Res (2020) 26(13):3345–59. doi: 10.1158/1078-0432.Ccr-19-3211 PMC7334097 32193227

[52] Wouters MCA Nelson BH. Prognostic significance of tumor-infiltrating b cells and plasma cells in human cancer. Clin Cancer Res: Off J Am Assoc Cancer Res (2018) 24(24):6125–35. doi: 10.1158/1078-0432.Ccr-18-1481 30049748

[53] Liu X Wu S Yang Y Zhao M Zhu G Hou Z. The prognostic landscape of tumor-infiltrating immune cell and immunomodulators in lung cancer. Biomed Pharmacother Biomed Pharmacother (2017) 95:55–61. doi: 10.1016/j.biopha.2017.08.003 28826097

[54] Hennequin A Derangère V Boidot R Apetoh L Vincent J Orry D. Tumor infiltration by tbet+ effector T cells and Cd20+ b cells is associated with survival in gastric cancer patients. Oncoimmunology (2016) 5(2):e1054598. doi: 10.1080/2162402x.2015.1054598 27057426 PMC4801425

[55] Brunner SM Itzel T Rubner C Kesselring R Griesshammer E Evert M. Tumor-infiltrating b cells producing antitumor active immunoglobulins in resected hcc prolong patient survival. Oncotarget (2017) 8(41):71002–11. doi: 10.18632/oncotarget.20238 PMC5642613 29050338

[56] Jensen TO Schmidt H Møller HJ Donskov F Høyer M Sjoegren P. Intratumoral neutrophils and plasmacytoid dendritic cells indicate poor prognosis and are associated with Pstat3 expression in ajcc stage I/Ii melanoma. Cancer (2012) 118(9):2476–85. doi: 10.1002/cncr.26511 21953023

[57] Kießler M Plesca I Sommer U Wehner R Wilczkowski F Müller L. Tumor-infiltrating plasmacytoid dendritic cells are associated with survival in human colon cancer. J Immunother Cancer (2021) 9(3):e001813. doi: 10.1136/jitc-2020-001813 33762320 PMC7993360

[58] Shang B Liu Y Jiang SJ Liu Y. Prognostic value of tumor-infiltrating Foxp3+ regulatory T cells in cancers: A systematic review and meta-analysis. Sci Rep (2015) 5:15179. doi: 10.1038/srep15179 26462617 PMC4604472

[59] Feng J Dai W Mao Y Wu L Li J Chen K. Simvastatin re-sensitizes hepatocellular carcinoma cells to sorafenib by inhibiting hif-1α/Ppar-Γ/Pkm2-Mediated glycolysis. J Exp Clin Cancer Res: CR (2020) 39(1):24. doi: 10.1186/s13046-020-1528-x 32000827 PMC6993409

[60] Wang ST Ho HJ Lin JT Shieh JJ Wu CY. Simvastatin-induced cell cycle arrest through inhibition of Stat3/Skp2 axis and activation of ampk to promote P27 and P21 accumulation in hepatocellular carcinoma cells. Cell Death Dis (2017) 8(2):e2626. doi: 10.1038/cddis.2016.472 28230855 PMC5386458

[61] Relja B Meder F Wang M Blaheta R Henrich D Marzi I. Simvastatin modulates the adhesion and growth of hepatocellular carcinoma cells *Via* decrease of integrin expression and rock. Int J Oncol (2011) 38(3):879–85. doi: 10.3892/ijo.2010.892 21206971

[62] Shirley M. Encorafenib and binimetinib: First global approvals. Drugs (2018) 78(12):1277–84. doi: 10.1007/s40265-018-0963-x 30117021

[63] Kim JW Lee KH Kim JW Suh KJ Nam AR Bang JH. Enhanced antitumor effect of binimetinib in combination with capecitabine for biliary tract cancer patients with mutations in the Ras/Raf/Mek/Erk pathway: Phase ib study. Br J Cancer (2019) 121(4):332–9. doi: 10.1038/s41416-019-0523-5 PMC6738070 31312030

[64] Zhou Q Wong CH Lau CP Hui CW Lui VW Chan SL. Enhanced antitumor activity with combining effect of mtor inhibition and microtubule stabilization in hepatocellular carcinoma. Int J Hepatol (2013) 2013:103830. doi: 10.1155/2013/103830 23509629 PMC3590758

[65] Tang Y Zhang Y Hu X. Identification of potential hub genes related to diagnosis and prognosis of hepatitis b virus-related hepatocellular carcinoma *Via* integrated bioinformatics analysis. BioMed Res Int (2020) 2020:4251761. doi: 10.1155/2020/4251761 33376723 PMC7744201

[66] Zhang Y Tang Y Guo C Li G. Integrative analysis identifies key mrna biomarkers for diagnosis, prognosis, and therapeutic targets of hcv-associated hepatocellular carcinoma. Aging (2021) 13(9):12865–95. doi: 10.18632/aging.202957 PMC8148482 33946043

[67] Tang Y Guo C Yang Z Wang Y Zhang Y Wang D. Identification of a tumor immunological phenotype-related gene signature for predicting prognosis, immunotherapy efficacy, and drug candidates in hepatocellular carcinoma. Front Immunol (2022) 13:862527. doi: 10.3389/fimmu.2022.862527 35493471 PMC9039265

[68] Li G Xu W Zhang L Liu T Jin G Song J. Development and validation of a cimp-associated prognostic model for hepatocellular carcinoma. EBioMedicine (2019) 47:128–41. doi: 10.1016/j.ebiom.2019.08.064 PMC6796541 31492561

[69] Dai X Jiang W Ma L Sun J Yan X Qian J. A metabolism-related gene signature for predicting the prognosis and therapeutic responses in patients with hepatocellular carcinoma. Ann Trans Med (2021) 9(6):500. doi: 10.21037/atm-21-927 PMC8039687 33850897

[70] Rao J Wu X Zhou X Deng R Ma Y. Development of a prognostic model for hepatocellular carcinoma using genes involved in aerobic respiration. Aging (2021) 13(9):13318–32. doi: 10.18632/aging.203021 PMC8148449 33903282

