# Supplementary figures and images for: M2-like tumor-associated macrophage-related biomarkers to construct a novel prognostic signature, reveal the immune landscape, and screen drugs in hepatocellular carcinoma
Source: Front Immunol. 2022 Sep 13;13:994019. doi: 10.3389/fimmu.2022.994019 (PMC9513313; doi:10.3389/fimmu.2022.994019)

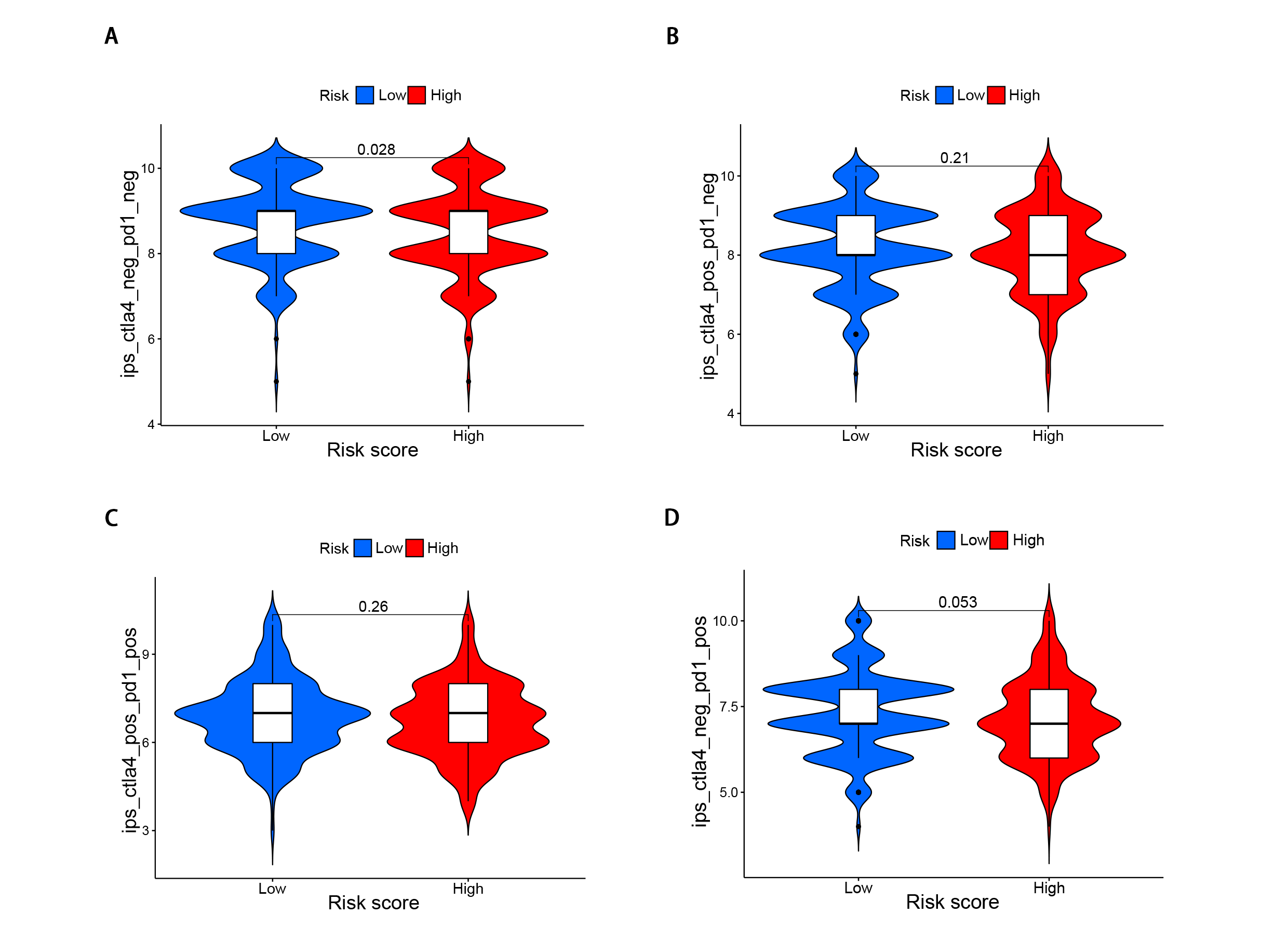

Supplement: Supplementary Figure 1 — (A–D) Scores for immunotherapy from the TCIA database. TCIA, The Cancer Immunome Atlas. [file Image_1.tif]

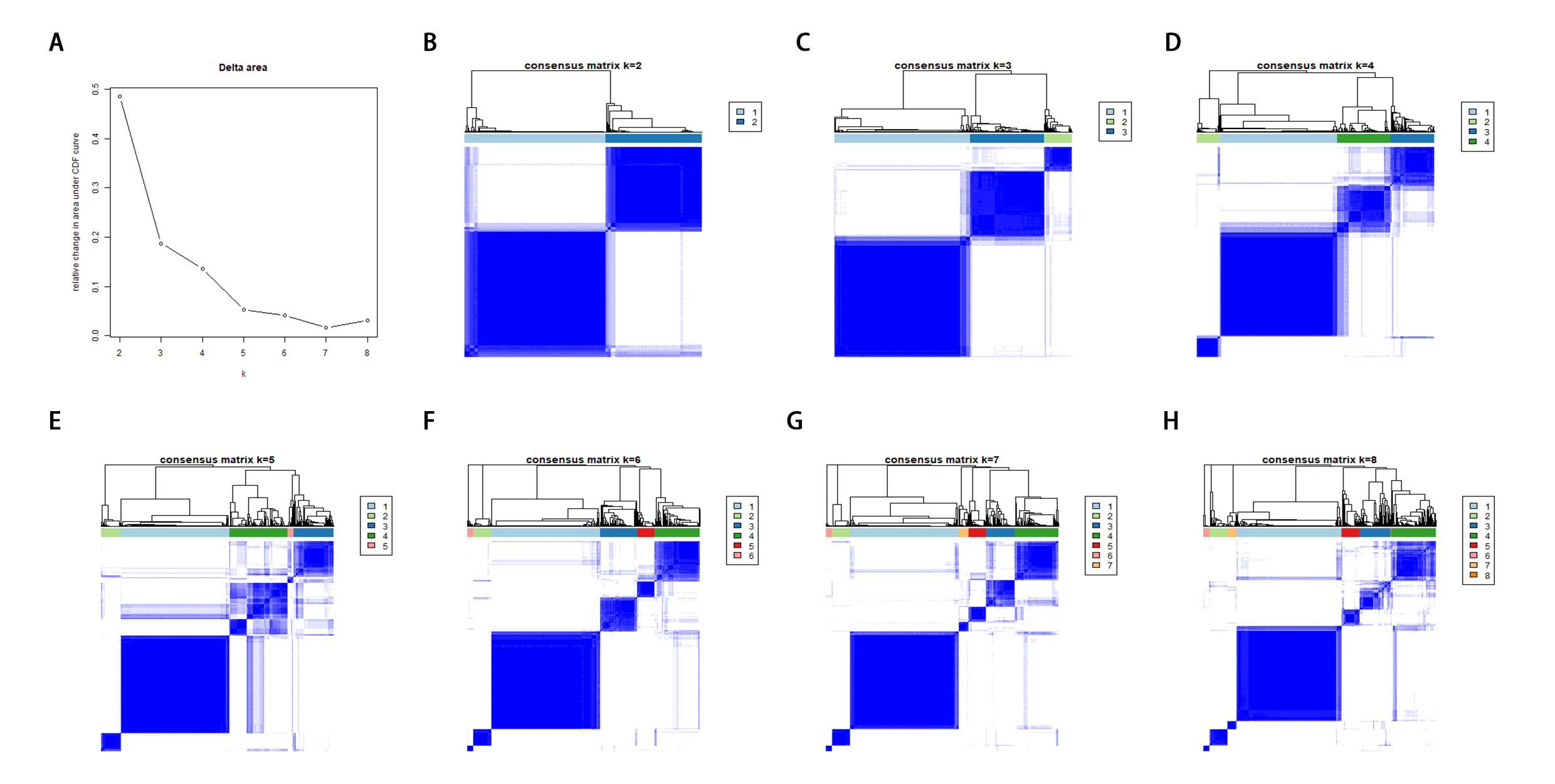

Supplement: Supplementary Figure 2 — (A–H) The results of consensus clustering. [file Image_2.tif]

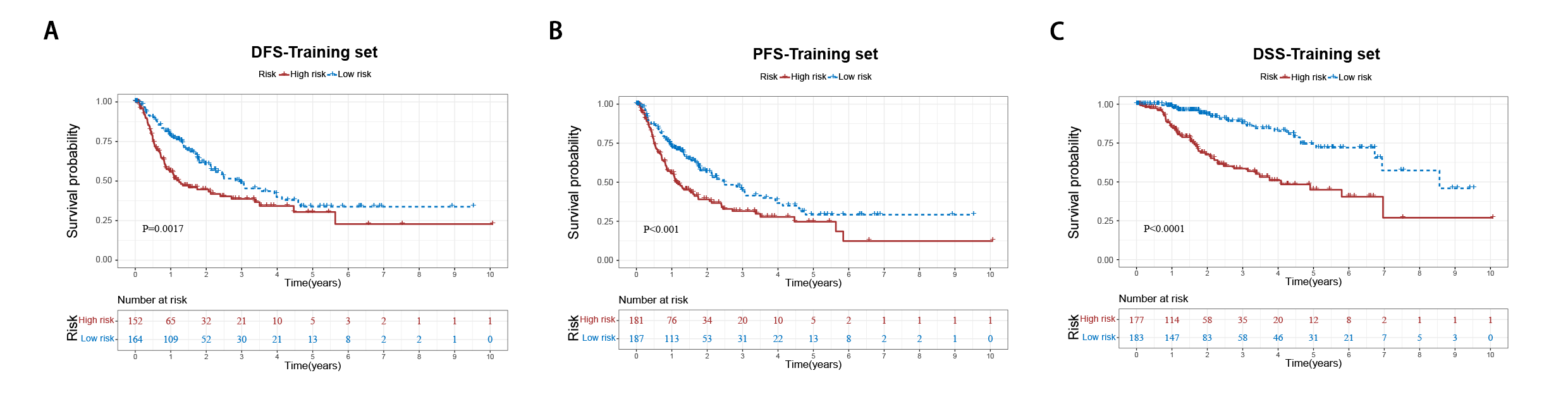

Supplement: Supplementary Figure 3 — Kaplan–Meier survival curves of risk score for DFS (A), PFS (B), and DSS (C) in TCGA-LIHC cohort. DFS, disease-free survival; PFS, progression-free survival; DSS, disease-specific survival. [file Image_3.tif]
