# Supplementary material for: M2-like tumor-associated macrophage-related biomarkers to construct a novel prognostic signature, reveal the immune landscape, and screen drugs in hepatocellular carcinoma
Source: Front Immunol. 2022 Sep 13;13:994019. doi: 10.3389/fimmu.2022.994019 (PMC9513313; doi:10.3389/fimmu.2022.994019)
Supplement: Supplementary Table 1 — Demographic information of hepatocellular carcinoma patients in this study. [file Table_1.docx]

**Supplementary Tables**

**Supplementary Table S1.** Demographic information of hepatocellular carcinoma patients in this study.

| Characteristics | TCGA-LIHC  (Training set) | GSE76427 | ICGC-LIRI-JP | Merged dataset  (Test set) |
| --- | --- | --- | --- | --- |
| Total | 368 | 115 | 232 | 347 |
| Gender |  |  |  |  |
| Male | 249 | 93 | 171 | 264 |
| Female | 119 | 22 | 61 | 83 |
| Age |  |  |  |  |
| ≤65 | 230 | 65 | 90 | 155 |
| ＞65 | 138 | 50 | 142 | 192 |
| TNM stage |  |  |  |  |
| I | 172 | 55 | 36 | 91 |
| II | 85 | 35 | 106 | 141 |
| III | 83 | 21 | 71 | 92 |
| VI | 4 | 3 | 19 | 22 |
| Unknown | 24 | 1 | - | 1 |
| Grade |  |  |  |  |
| G1 | 55 | - | - | - |
| G2 | 176 | - | - | - |
| G3 | 120 | - | - | - |
| G4 | 12 | - | - | - |
| Unknown | 5 | - | - | - |
| T stage |  |  |  |  |
| T1 | 182 | - | - | - |
| T2 | 92 | - | - | - |
| T3 | 78 | - | - | - |
| T4 | 13 | - | - | - |
| Unknown/TX | 3 | - | - | - |
| N stage |  |  |  |  |
| N0 | 250 | - | - | - |
| N1 | 4 | - | - | - |
| Unknown/NX | 114 | - | - | - |
| M stage |  |  |  |  |
| M0 | 265 | - | - | - |
| M1 | 3 | - | - | - |
| Unknown/MX | 100 | - | - | - |

**Supplementary Table S2.** Clinicopathological information of 15 patients with hepatocellular carcinoma in this study.

| Patients | Age | Gender | Tumor size(cm) | T stage | N stage | M stage |
| --- | --- | --- | --- | --- | --- | --- |
| 1 | 50 | Male | 2.4*2.0 | T1a | N0 | M0 |
| 2 | 57 | Male | 3.8*3.5 | T1a | N0 | M0 |
| 3 | 48 | Male | 5.6*5.7 | T3b | N0 | M0 |
| 4 | 57 | Male | 3.0*3.0 | T1a | N0 | M0 |
| 5 | 64 | Male | 1.0*1.1 | T1b | N0 | M0 |
| 6 | 44 | Male | 4.0*3.0 | T2 | N0 | M0 |
| 7 | 62 | Male | 5.1*3.5 | T2 | N0 | M0 |
| 8 | 64 | Female | 10.0*8.0 | T4 | N0 | M0 |
| 9 | 61 | Female | 7.9*7.5 | T2 | N0 | M0 |
| 10 | 49 | Female | 3.0*2.0 | T1a | N0 | M0 |
| 11 | 52 | Female | 3.2*2.4 | T1b | N0 | M0 |
| 12 | 51 | Female | 5.8*5.0 | T1b | N0 | M0 |
| 13 | 67 | Female | 9.6*8.8 | T1b | N0 | M0 |
| 14 | 46 | Female | 1.1*1.6 | T1a | N0 | M0 |
| 15 | 43 | Female | 6.2*5.7 | T3 | N0 | M0 |

**Supplementary Table S3.** The primers sequences used in this study.

|  | Primers sequence (5′-3′) | |
| --- | --- | --- |
|  | Forward primer | Reverse primer |
| PDLIM3 | CTCACCTCAAAACGAGCCCA | CATGGACTTTCGTCACCGGA |
| PAM | TACCACCAGACCCGTAGTTCC | GTTTAGGTGTAACCCCAGGCA |
| PDLIM7 | CAGAGCCGCACCTCCATTG | TGGTGACACACGGGAGTCT |
| FSCN1 | CACACGGGCAAGTACTGGAC | TCTGAGTCCCCTGCTGTCTC |
| LGALS3 | GGAGAGGGAATGATGTTGCCT | TCCTGCTTCGTGTTACACACA |
| GAPDH | CGGATTTGGTCGTATTGGG | CGCTCCTGGAAGATGGTGAT |

**Supplementary Table S4.** List of 405 genes from the red module of the WGCNA analysis. WGCNA, weighted gene co-expression network analysis.

| SAMD11 | CXCL12 | MYO10 | CRISPLD2 | EHD3 | KRT7 | AEBP1 | HAMP |
| --- | --- | --- | --- | --- | --- | --- | --- |
| MXRA8 | ALOX5 | LIFR | PIEZO1 | EFEMP1 | IGFBP6 | ELN | HSPB6 |
| MEGF6 | ARID5B | C7 | DPEP1 | ANTXR1 | ARHGEF25 | SEMA3C | CLIP3 |
| PDPN | EGR2 | ESM1 | NXN | THNSL2 | MSRB3 | HGF | PPP1R14A |
| FBLIM1 | PALD1 | GPX8 | ABR | FHL2 | LUM | COL1A2 | ZFP36 |
| EPHA2 | UNC5B | MAP1B | SPNS2 | RND3 | DCN | LRRC17 | LTBP4 |
| MFAP2 | CHST3 | F2RL2 | NLGN2 | CYBRD1 | ELK3 | LAMB1 | AXL |
| PLA2G5 | DDIT4 | F2R | PMP22 | FRZB | TMEM119 | CAV1 | FOSB |
| ID3 | PLAU | CRHBP | MFAP4 | COL3A1 | TRPV4 | PTN | EHD2 |
| SLC9A1 | ACTA2 | VCAN | TMEM97 | COL5A2 | POSTN | TRIM24 | PPP1R15A |
| MAP3K6 | CH25H | EDIL3 | RNF135 | NRP2 | KCTD12 | NOS3 | RCN3 |
| FCN3 | MYOF | PAM | MYO1D | IGFBP5 | ITGBL1 | PTPRN2 | CLEC11A |
| TINAGL1 | MARVELD1 | AP3S1 | CCL2 | GPBAR1 | COL4A1 | DEFB1 | MYADM |
| COL16A1 | TLX1 | SLC12A2 | ACACA | DES | COL4A2 | LOXL2 | CDC42EP5 |
| COL8A2 | CALHM2 | CXCL14 | FZD2 | KCNE4 | MMP14 | DPYSL2 | CPXM1 |
| HEYL | ADRA2A | EGR1 | NGFR | SERPINE2 | NFATC4 | SCARA5 | RASSF2 |
| PODN | CPXM2 | TMEM173 | SGCA | COL6A3 | LGALS3 | RNF122 | JAG1 |
| TACSTD2 | CTBP2 | SPRY4 | COL1A1 | LMCD1 | LTBP2 | FGFR1 | KIZ |
| NEXN | GALNT18 | SH3RF2 | MTMR4 | FBLN2 | PGF | PLAT | FOXA2 |
| CYR61 | DKK3 | DPYSL3 | TBX2 | ITGA9 | FOS | SULF1 | THBD |
| F3 | SPON1 | PDGFRB | MRC2 | PTH1R | JDP2 | STMN2 | ID1 |
| COL11A1 | FIBIN | SPARC | C17orf58 | IFRD2 | TGFB3 | GEM | FOXS1 |
| GSTM4 | PAMR1 | SLIT3 | TTYH2 | HYAL3 | VASH1 | TSPYL5 | PROCR |
| MOV10 | FJX1 | GABRP | TMEM104 | BAP1 | FBLN5 | CTHRC1 | MYL9 |
| OLFML3 | C11orf96 | PDLIM7 | CDR2L | ARHGEF3 | BDKRB2 | COLEC10 | WISP2 |
| ECM1 | TP53I11 | ADAMTS2 | TIMP2 | KCTD6 | JAG2 | NOV | SULF2 |
| CTSK | CHST1 | GFPT2 | C1QTNF1 | EPHA3 | THBS1 | COL14A1 | PTGIS |
| NES | CREB3L1 | FOXC1 | LRRC45 | COL8A1 | FBN1 | CCL19 | SNAI1 |
| ACKR1 | VWCE | EDN1 | FN3KRP | FILIP1L | MYO1E | CCL21 | PMEPA1 |
| FCER1A | EFEMP2 | MCUR1 | METRNL | CCDC80 | TPM1 | RECK | STMN3 |
| OLFML2B | FOSL1 | ID4 | RAB31 | FSTL1 | RASL12 | ANXA1 | CLIC6 |
| RGS4 | CD248 | B3GALT4 | TUBB6 | ADCY5 | LOXL1 | GAS1 | COL6A1 |
| PBX1 | B4GAT1 | GLO1 | ONECUT2 | HEG1 | ISLR | ROR2 | COL6A2 |
| DPT | LRFN4 | MDFI | FSTL3 | PLXND1 | CSPG4 | OGN | SCARF2 |
| SELP | ARHGEF17 | RCAN2 | PALM | PCOLCE2 | CEMIP | ASPN | SUSD2 |
| KIAA0040 | LRRC32 | COL12A1 | TBXA2R | LXN | MFGE8 | LPAR1 | GGT5 |
| FAM129A | PRSS23 | LAMA4 | PTPRS | CLDN11 | IQGAP1 | TNC | PDGFB |
| FMOD | ENDOD1 | COL10A1 | CLEC4G | P3H2 | SLCO3A1 | ANGPTL2 | A4GALT |
| PRELP | MMP7 | NCOA7 | ADAMTS10 | APOD | CHSY1 | FAM129B | FAM19A5 |
| CAPN2 | PDGFD | LAMA2 | SLC44A2 | AFAP1 | CACNA1H | CERCAM | MXRA5 |
| C1orf198 | TAGLN | CTGF | CNN1 | HTRA3 | MYH11 | LAMC3 | SCML1 |
| AKT3 | MCAM | MOXD1 | JUNB | SOD3 | GPRC5B | AIF1L | RAI2 |
| PXDN | THY1 | FNDC1 | SYDE1 | SHISA3 | TMEM159 | ADAMTS13 | SRPX |
| COLEC11 | NRGN | SMOC2 | NOTCH3 | PDGFRA | MMP2 | ADAMTSL2 | TIMP1 |
| CYS1 | P3H3 | THBS2 | TPM4 | IGFBP7 | CDH11 | COL5A1 | EFNB1 |
| VSNL1 | MGP | PDGFA | KLF2 | EDNRA | RRAD | OLFM1 | SRPX2 |
| KCNS3 | RERG | FSCN1 | ARRDC2 | SFRP2 | B3GNT9 | PTGDS | ARMCX1 |
| TTC32 | PDE3A | INMT | ISYNA1 | NPY1R | FHOD1 | NPDC1 | ARMCX2 |
| EMILIN1 | TUBA1A | AQP1 | COMP | HAND2 | TPPP3 | NRARP |  |
| LBH | GRASP | SFRP4 | KCTD15 | PDLIM3 | IL34 | CCDC3 |  |
| PLXDC2 | FLNA | VIM | BGN | FZD8 | SMIM10 | TCEAL3 |  |

**Supplementary Table S5.** List of 2047 TAM marker genes obtained from scRNA-seq data analysis

| AIF1 | CLIC1 | NCF4 | ITGA10 | ARL4C | FAM162B | PRNP | ACSL1 |
| --- | --- | --- | --- | --- | --- | --- | --- |
| FCER1G | SLC9A3R2 | CARD16 | HGSNAT | PTPN6 | ENAH | C1R | C11orf58 |
| LST1 | CAPZB | COL4A2 | SHKBP1 | KCNMA1 | SNHG7 | MT1E | NCOA7 |
| MS4A7 | SYNPO | CYR61 | RND1 | ACTB | TGFBR3 | NAGA | SSTR2 |
| CPVL | MPRIP | ALDH3B1 | GSTA1 | BTK | RNF13 | MAST4 | CP |
| CLEC7A | MYCT1 | RPL34 | AQP3 | LITAF | CD3D | VMA21 | TMEM9B |
| LGALS2 | ACER3 | LUCAT1 | RANBP2 | C9orf72 | ACTR10 | SLC25A5 | RAB2A |
| MS4A6A | CYYR1 | AP2S1 | ENG | CXCL3 | OTOA | ADGRL4 | GADD45GIP1 |
| MNDA | RAPGEF5 | ID2 | CD3E | BASP1 | RUNX1T1 | ADIRF | HSD17B6 |
| IL1B | SLC44A2 | MGP | ZNF667-AS1 | AREG | CNOT6L | MT2A | STK4 |
| TYROBP | EIF3H | ITM2C | DYSF | SGK1 | HDLBP | PQLC3 | BTF3L4 |
| C1orf162 | TMEM59 | SNX10 | POLR2F | TYMP | VPS29 | STK17B | BOLA3 |
| LY86 | RUNX1 | RNF144B | LOXL2 | STXBP2 | ZYX | ADAMTS9 | GNAI3 |
| MPEG1 | ZFYVE16 | ATP6V0E1 | NT5C3A | MYO1F | ASXL1 | ARHGAP4 | ANGPTL3 |
| HLA-DQA1 | FBXO32 | BATF3 | TDO2 | GK | HDAC7 | ARL8B | TIPARP |
| LYZ | TAOK3 | GDI2 | MYH9 | CIITA | MED21 | AHR | BACH1 |
| HLA-DMB | BLOC1S1 | ZNF331 | PELO | RAB32 | ARAP1 | RP11-11N9.4 | SPTSSA |
| ITGAX | BMP2K | ANXA5 | RSRC2 | GAPT | PON2 | RASSF3 | AHI1 |
| SPI1 | CHN2 | CFL1 | CLNS1A | NINJ1 | TBX2-AS1 | SNN | SLC12A2 |
| HLA-DQB1 | PCSK7 | PTP4A3 | TMEM106B | EMP3 | AC013461.1 | NPDC1 | ATRX |
| IGSF6 | WASL | METRNL | ITGA7 | ITGB2-AS1 | COMMD9 | RPS5 | PGAP1 |
| C5AR1 | COL15A1 | RGS2 | ERGIC2 | SLC43A2 | VASN | SH3BGRL | TPPP3 |
| CSF1R | LAMTOR2 | LGALS3 | CPM | TPP1 | CYB5A | PLAT | CA2 |
| CFD | PCGF2 | SLC25A4 | ROBO4 | MFSD1 | SLCO2A1 | MYL9 | COL1A1 |
| BCL2A1 | MYO1C | SELPLG | UVRAG | MXD1 | C4orf32 | P2RY14 | IKZF3 |
| MSR1 | TMEM88 | TFPI | EIF3F | BID | MIF4GD | TPM2 | ATF4 |
| CD68 | GNAI1 | PID1 | GIMAP6 | PTPRC | PXDN | NUPR1 | LINC00152 |
| CYBB | FUCA1 | RPL30 | NFIC | WAS | RBM3 | RAB13 | ZNF638 |
| PILRA | HIST1H2AC | STOM | BNIP3L | MAP3K8 | BAD | RPL5 | CACUL1 |
| HCK | PBX1 | GLIPR2 | NDUFS2 | NCKAP1L | SOD1 | EIF4A2 | ARID4B |
| CLEC12A | EIF3K | CFLAR | AAMDC | SHTN1 | ATP6V1G1 | VASP | IRF3 |
| MS4A4A | KTN1 | TNFAIP8 | SNX29 | ATP5E | PLOD1 | SUPT4H1 | 2-Sep |
| PLAUR | IL10RB | NABP1 | RRAS | CMTM6 | GALNT18 | PRSS23 | RBBP7 |
| CD86 | LRP1 | EIF1 | TRIM56 | FAM49A | IGHG1 | DERL3 | MRPL17 |
| CLEC10A | PCBP1 | LINC00877 | ARRDC1 | SIGLEC10 | EVA1C | ADGRF5 | IGF2 |
| VSIG4 | VGLL4 | CALM2 | GTF2B | BCAT1 | ERP29 | RHOB | THOC6 |
| CSF2RA | ANXA6 | TWF2 | DAPK3 | RP5-1171I10.5 | AGRN | C14orf2 | AIDA |
| CLEC4A | SPATS2 | COX7A1 | GFOD1 | GMFG | MRPS31 | RPS28 | UQCRFS1 |
| HLA-DRB5 | TIE1 | TSC22D1 | SIPA1L1 | SLC25A6 | GIMAP7 | RPL7A | NKTR |
| LILRB2 | KLF9 | APP | PPP1R12A | AVPI1 | HIST1H2BG | GNG5 | SLFN5 |
| C15orf48 | CALU | TNF | JMJD1C | C10orf54 | MFSD14B | RGS5 | SDSL |
| FCN1 | POU2F2 | RPS20 | JADE1 | DRAM2 | WTAP | NACA | CLK1 |
| HLA-DPB1 | ODF2L | NEAT1 | DGKH | LYN | UBE2E2 | RBPJ | TFR2 |
| FGL2 | ACTG1 | PKM | GUCY1A3 | RBM47 | DBNL | PPFIBP1 | ACAP1 |
| FAM26F | IGHM | SFT2D1 | SCPEP1 | RPL11 | ACAT1 | CSRP2 | NDUFB9 |
| C1QC | STC1 | RPL37 | RTN4 | DPEP2 | RALBP1 | PLXNC1 | CDC42EP2 |
| NLRP3 | TAGLN | NFE2L2 | CD46 | TSPO | PRKD2 | LGMN | ABCD4 |
| CD83 | ENPEP | LAMTOR4 | RASAL2 | NFKBIA | CLEC2D | WBP5 | STK40 |
| RNASE6 | UQCR10 | IL4I1 | BNIP3 | CAV1 | RASD1 | CD1D | COMMD4 |
| CSTA | CRISPLD2 | ATG3 | SDC2 | RPS12 | FAM96A | BRK1 | CEBPD |
| ITGB2 | MMRN2 | 7-Sep | SOCS3 | RPS16 | THSD7A | DCN | IGHA1 |
| IFI30 | DOCK6 | RPL15 | SPNS1 | CTSC | SLC39A7 | LTA4H | GCSH |
| HLA-DPA1 | MPDZ | C20orf27 | AC058791.1 | SAMSN1 | OAF | RBP1 | CYSTM1 |
| S100A9 | PPP1R18 | LRRFIP1 | CYLD | DAPK1 | ADGRA2 | ITGA6 | HNRNPR |
| FCGR2A | SRP14 | PKIG | SMARCE1 | RPS17 | TCEA1 | ROGDI | RNF167 |
| PLEK | IRS2 | PLXDC2 | FAM214A | RPS3A | WSB1 | IL13RA1 | CCDC82 |
| NCF2 | SYNE1 | MAP1B | DDR2 | CYTH4 | PIK3C2A | CDC42SE1 | NDUFS5 |
| IL18 | RSBN1L | RABAC1 | STX4 | RPL26 | MTRNR2L10 | EIF3E | GZMA |
| TREM1 | CREB3L2 | PLAC9 | 1-Sep | ATP6V1F | SYPL1 | EIF3L | TM7SF2 |
| HLA-DRA | COX4I2 | SNX3 | SMDT1 | ARPC5 | DAD1 | PTK2 | BAAT |
| HLA-DMA | SPAG4 | NR4A2 | ETHE1 | CALD1 | YIPF3 | TMEM47 | RP11-138A9.1 |
| C1QA | OPN3 | MAP1LC3A | MID1IP1 | MCL1 | BDH2 | CFH | HULC |
| FCGR3A | FGD4 | GNG11 | ST6GALNAC6 | NPL | FAM114A1 | ELF1 | SMC4 |
| GPR34 | C9orf3 | RPS6KA1 | IFT43 | IL1R2 | CREB5 | TM6SF1 | DYNC1I2 |
| HLA-DRB1 | MAP3K7CL | RPL13A | HPS1 | H3F3A | ERRFI1 | EPSTI1 | NSA2 |
| FPR3 | MAP3K2 | ATP6V0D1 | PDGFA | RIPK2 | PRKAG2-AS1 | MDK | TCEAL4 |
| RGS10 | MTPN | PRKCDBP | UBA2 | RPL39 | MGEA5 | CD34 | FGFR1OP2 |
| TNFSF13B | GRK5 | S100A16 | CCSER2 | GCA | ROCK2 | SNX2 | IRF1 |
| JAML | TSPAN9 | RRBP1 | TMEM2 | RPL18A | C12orf65 | IL10RB-AS1 | WARS |
| LILRB4 | ADH5 | MBP | ZBTB43 | CCL4L2 | CTGF | ISYNA1 | HMGCS2 |
| LRRC25 | KDR | TRAF1 | CHCHD10 | NAGK | KLHL23 | CTTN | BTG3 |
| AOAH | PLEKHB2 | COL6A2 | RP11-160E2.6 | CSTB | CDC42EP4 | ARHGEF12 | LEAP2 |
| SLC7A7 | FAM3C | NFKBIE | YWHAQ | GLUL | ZFYVE21 | CLEC2B | POLR1D |
| OLR1 | SVIP | ZFAS1 | SSR1 | SLC11A1 | IGKV1-5 | GPCPD1 | CD9 |
| CSF3R | PLD3 | DENND6B | CLU | RPL27A | LMOD1 | ANGPT2 | LDLR |
| CXCL8 | C5orf24 | MYD88 | ARHGEF7 | RPLP1 | SLC29A1 | CRIM1 | TMEM176A |
| CD74 | VPS35 | ITGA1 | DENND1B | MMP19 | PDGFD | CD99 | ABHD5 |
| CST3 | EPS8 | EGR1 | ALCAM | RAP2B | ATP8B1 | AP1B1 | C12orf75 |
| PLA2G7 | RCN2 | FILIP1 | GAS6 | RPS23 | GNS | AQP1 | UGCG |
| CXCL16 | SLC40A1 | ALDH2 | ACE | LIMD2 | COX4I1 | PIK3AP1 | GCH1 |
| CTSS | PPP1R12B | HSP90AA1 | UBR1 | NAAA | TNFRSF10D | EGFL7 | TACC1 |
| C1QB | FKBP10 | TPM1 | FAM127A | SERPINB1 | TSHZ2 | UBC | CCDC167 |
| THEMIS2 | SOD3 | RPS27A | KLC1 | ABRACL | CCNI | SKAP2 | TUBA1C |
| HAVCR2 | CCDC88A | PDE4B | OXSR1 | FMNL1 | PLK3 | ACTR3 | SPON2 |
| TLR2 | FAM174A | SERPINA1 | NQO1 | PFN1 | GPAT2 | CALM1 | UBE2F |
| CD33 | F2R | M6PR | LSM14A | IL32 | CD200 | ICAM1 | REV3L |
| IRF8 | FSTL1 | LBH | GOLGA4 | TGIF1 | ADH1B | PODXL | BLOC1S2 |
| STX11 | KRT18 | TIMP3 | IGFBP3 | ATP6V1B2 | NMD3 | UBE2D3 | EIF4H |
| HLA-DOA | RPS18 | PPIC | ANPEP | DMXL2 | KIAA0355 | MX2 | SERPINA6 |
| HMOX1 | C11orf96 | SARAF | GJC1 | BLVRB | GOLGB1 | FRZB | FKBP2 |
| CD163 | RBFOX2 | TCF4 | FAM107A | PFDN5 | AIF1L | EBF1 | DCTN6 |
| GPR183 | EFEMP2 | MARCKS | SLC8B1 | DSE | PTMS | RAB7A | SULT1A1 |
| CLEC4E | C7orf73 | SNX8 | FIS1 | SLC8A1 | RB1 | C10orf10 | SON |
| PYCARD | MSI2 | NFIA | TEK | PAK1 | COL14A1 | DNAJB1 | TGM2 |
| 1-Mar | ATP6AP1 | NR2F2 | ADGRG1 | RASSF5 | IP6K2 | TNS1 | OLA1 |
| ADGRE2 | NT5C3B | MOB1A | NSRP1 | ATP6V0B | TMEM233 | KRT10 | ACAA1 |
| FTH1 | CAP1 | AK1 | LTBP1 | LINC00936 | DDX24 | SNCG | CHORDC1 |
| CD1C | INSR | PIK3R3 | EIF3M | RHOG | ECHDC2 | NUDT1 | GPATCH8 |
| NPC2 | JAM2 | KIAA0930 | RSF1 | RPL23 | APLNR | SDPR | ZSWIM7 |
| CFP | GNG12 | PDE4A | PPM1K | FBP1 | VWA1 | SEPW1 | SKP1 |
| PTAFR | GLO1 | ETV3 | CSPG4 | TNFAIP2 | ZCCHC11 | FXYD1 | KRTCAP2 |
| RNASET2 | IGKV1-12 | SMTN | SNRPN | CELF2 | CFAP20 | CPE | STK38L |
| CAPG | PDK4 | SIRPA | ARID3A | RPLP2 | KLF6 | NBPF14 | HRSP12 |
| TREM2 | TGFBR2 | TM4SF1 | MOB2 | EFHD2 | KALRN | YPEL5 | DPYSL2 |
| PHACTR1 | GNAQ | CMTM7 | SERPING1 | GAS7 | TMEM14A | SDC1 | TNFSF10 |
| LINC01272 | SCNM1 | MTUS1 | SCOC | NAP1L1 | MRPS26 | SYNGR2 | CYP3A5 |
| F13A1 | TRAC | PLS3 | C4BPB | RPS14 | ARHGEF17 | HIST1H1C | ARF4 |
| KYNU | SELENBP1 | THY1 | DYNLL1 | TKT | MRPL2 | CD320 | MED4 |
| ARRB2 | MIF | MFGE8 | NDRG1 | TGFBI | AKT3 | ST8SIA4 | STK17A |
| CD300A | PROCR | C8orf4 | RARA | CKLF | ANO1 | PLPP3 | APOM |
| GPX1 | CLMN | BLVRA | SRP9 | RASGEF1B | MAT2A | SERF2 | ZKSCAN1 |
| C3AR1 | AKR1C3 | NCOA4 | MYLIP | RPL19 | EIF2AK2 | RBFOX3 | ACSL3 |
| LAPTM5 | C19orf60 | LHFP | DNAJC4 | FAU | MMP2 | ECSCR | PDLIM7 |
| FOLR2 | SREK1 | CXorf40A | AHSA1 | RPS8 | PTPRN2 | APRT | NDUFA5 |
| FCGR1A | PLPP5 | PSTPIP2 | TMEM205 | SELM | PAIP1 | ECE1 | PHF20L1 |
| FTL | EEF1A1 | EIF4EBP1 | KNOP1 | GNA13 | IGHG4 | PLK2 | PXMP2 |
| SAMHD1 | WHSC1L1 | ACTR2 | LGALS3BP | AP001055.6 | YBX1 | CFL2 | SIGIRR |
| FAM49B | IFI6 | NCF1 | CYTH2 | EEF1B2 | EFHD1 | IFITM3 | TOM1 |
| CD300LF | PRMT1 | HBEGF | TFRC | CASP1 | ELK3 | NID1 | SNX5 |
| ALOX5 | JUP | S100A6 | CFB | VDR | FBN1 | EFNB2 | MIR4435-2HG |
| FPR1 | GFOD2 | MILR1 | TNFRSF4 | ATP1B3 | IL33 | RPS21 | ATP6V1H |
| RGS18 | GRB10 | RPS25 | RHOC | CRIP2 | ITFG1 | PLVAP | ITIH1 |
| CCL3 | GJA1 | MIR181A1HG | MAPK3 | RPL32 | ARPC5L | NCKAP1 | TMEM38B |
| EREG | MYH11 | REXO2 | CERS2 | CXCL2 | MECOM | STAT3 | HNRNPA2B1 |
| SLAMF8 | LSM6 | ADCY7 | GIMAP1 | TPT1 | ANKRD36 | ABI3 | ETV5 |
| PTPRE | 10-Sep | ZBTB16 | GUCY1B3 | CNN3 | RHOH | IFNGR2 | KIAA1033 |
| LAIR1 | MTRNR2L8 | PLPP1 | FRY | KCTD12 | TSPAN18 | IL6ST | MTRNR2L3 |
| VMO1 | COMMD6 | RPL4 | GAMT | RPL27 | TRIP6 | FNBP1 | COPS8 |
| G0S2 | B4GALT1 | RORA | PTRHD1 | HSPA1B | APH1A | CALCRL | EIF4E |
| TBXAS1 | PTPRM | ATP1B1 | TFF3 | S100B | AOC3 | AKAP12 | ANP32E |
| FAM105A | NRN1 | MAPKAPK3 | PSMB5 | SPTBN1 | MRPL23 | PGF | SERINC5 |
| LGALS9 | ERG | ITGB1 | PPP2R5E | SPARC | HECW2 | ETF1 | PSMD4 |
| OSM | B4GALT3 | ABCA1 | ACVRL1 | PTRF | KLHDC8B | DNAJB9 | APOBEC3C |
| PSAP | ARPC1A | PPP1R14A | HSPD1 | MT-CO1 | STAT1 | RPLP0 | 11-Sep |
| VAMP8 | NDFIP2 | GM2A | LCK | RENBP | CASKIN2 | RAMP3 | VKORC1 |
| SPINT2 | FAM167B | GJA4 | HMGB1 | RGS19 | ARL15 | CD47 | ETS2 |
| SRGN | SNAP23 | HSP90AB1 | FOS | RPL29 | UBE2D1 | GAA | UFC1 |
| OSCAR | COL6A1 | BGN | PLCE1 | SPARCL1 | TUBA1B | C1orf54 | LUC7L3 |
| UCP2 | VAMP2 | IRAK3 | CD3G | LACC1 | INTS6 | C1S | TAF1D |
| LSP1 | LACTB | FNIP2 | DYNLT3 | FKBP11 | GGT5 | LMAN1 | PLXND1 |
| LILRB3 | CFI | FBXO34 | FLNB | S100A4 | BMPR2 | BAZ1A | NAF1 |
| GRN | DAPP1 | THAP2 | SYAP1 | PDLIM1 | TERF2IP | RPS7 | NOSIP |
| P2RY13 | GSTM3 | CCDC85B | PPIB | LY96 | NBN | MYO6 | SEC14L1 |
| RP11-1143G9.4 | PCYOX1 | TJP1 | TRAPPC1 | PLCB2 | SH3PXD2A | CSK | CD2 |
| FCER1A | BBX | CCDC109B | FHL2 | SLC15A3 | RBP4 | SERPINI1 | MDH2 |
| GLIPR1 | FAM213A | ADAMTS1 | SPRED1 | GPR65 | TTR | PDGFRB | THBS1 |
| SCIMP | CTD-2336O2.1 | LIMS1 | ARL3 | RPS19 | HMCES | DYNC1LI2 | DYNLRB1 |
| AP1S2 | LIMS2 | IL6R | RNF152 | SMAP2 | INHBB | FKBP9 | PLXDC1 |
| IL1RN | HTRA1 | LDB2 | SUGT1 | RPS15 | FOXS1 | MYO1B | PCNP |
| CYBA | MRPL32 | ERLEC1 | GNL1 | SERP1 | ALB | MPHOSPH8 | RAB10 |
| SOD2 | NOSTRIN | EMP2 | SHISA5 | NOTCH2 | ISG20 | RBMS3 | MTRNR2L11 |
| TMSB4X | LRRC32 | PLAC8 | BSG | DBI | KLRB1 | HSPA1A | MYL12B |
| HCST | CCPG1 | PRDX4 | CEBPB | TMEM106A | SOX17 | NOTCH4 | HMGCS1 |
| S100A11 | BCAR1 | APOBEC3A | CORO1C | RPL14 | MYL6B | NOTCH3 | RNF213 |
| PLD4 | JCHAIN | CREBL2 | AADAC | UNC93B1 | SOX7 | TNFRSF17 | TM4SF4 |
| CD37 | MBNL2 | RPS13 | RBMS1 | CCL4 | SOS1 | DAB2 | IL7R |
| EPB41L3 | TXNDC15 | MAGED2 | ZNF581 | LGALS1 | PLG | KANK2 | TCIRG1 |
| OAZ1 | ATF5 | ZBTB20 | YAF2 | RPL22 | NAE1 | FLT1 | TNFRSF12A |
| CD72 | ARMCX3 | RAB11FIP1 | CHMP3 | C12orf57 | TSPAN13 | CD27 | LSM2 |
| ARPC1B | SGCB | RPL9 | AP3M2 | VCAN | EDNRA | CYB5R4 | TMOD3 |
| CD14 | LDHB | PKIB | SPG21 | HN1 | SKIL | NRROS | FSCN1 |
| RNF130 | NES | RPSA | AMZ2 | FERMT3 | PHF14 | LAMTOR1 | PSMD8 |
| FYB | HACD3 | MYLK | N4BP2L2 | RP11-214O1.3 | NBEAL1 | RPL10A | ZNF131 |
| S100A8 | CAMK2N1 | FERMT2 | MGST3 | SERPINB9 | CSRP1 | TXNIP | SLC2A6 |
| LCP1 | HSPA8 | OTUD1 | CPB2 | ATP5G2 | UFD1L | CDC42BPA | PHACTR2 |
| CLEC5A | RCAN2 | EFNA1 | PLA2G16 | SEC11A | ISCU | GUK1 | TBC1D9 |
| CLEC9A | COL3A1 | PPP1CB | CCT7 | HIGD2A | MEIS2 | SPTAN1 | RRAGA |
| RAB20 | MALL | FOSL2 | BTG2 | RPL13 | SMAGP | RGS3 | NUCB1 |
| PPT1 | OLFML2A | PALMD | TBC1D15 | CD55 | COL5A2 | RPL7 | VTI1B |
| CD300C | KIAA1462 | MOB3A | CCL14 | RPS15A | NMRK1 | S1PR1 | AHSG |
| CNPY3 | SUN1 | ADAMTS4 | CES2 | ATP2B1 | PSME2 | TCOF1 | UBXN4 |
| GPAT3 | RTF1 | ANXA1 | CTSL | ITM2A | AES | PTPRG | LAP3 |
| PLBD1 | FAM63B | RPL18 | CCDC102B | RPL21 | RNF5 | MSRB3 | UTRN |
| CD1E | FABP5 | RPL38 | EIF2S3 | IGFBP7 | STX7 | MAGI1 | DPP7 |
| SLC1A3 | RPL37A | COBLL1 | SMARCC2 | NFKB1 | PMEPA1 | CARHSP1 | EDEM1 |
| FGR | PDLIM3 | RASSF4 | SMC3 | MGAT1 | PLN | KCNK6 | ASL |
| GNA15 | FABP4 | NUCB2 | MLXIPL | PGLS | EID1 | FDPS | TMCO1 |
| SAT1 | NUAK1 | 4-Sep | ZFAND2A | SH2B3 | MYO10 | IQGAP2 | ADAM15 |
| LILRA2 | POMP | DNAJB4 | PHF1 | UBB | NRP1 | GPRC5C | IGKC |
| TFEC | CSNK1E | SORBS2 | RSL1D1 | ADAP2 | NUCKS1 | COL1A2 | TCP1 |
| ITGAM | CLIC4 | MTMR14 | PRKCH | SLC25A19 | RBP7 | ASS1 | HP1BP3 |
| ZNF385A | HOXB2 | RBPMS | UBXN1 | SELL | MCOLN1 | LAMB2 | FGL1 |
| PRAM1 | PCBP2 | MPP1 | NDRG2 | NFIB | GNAI2 | GSTO1 | NDUFA6 |
| FGD2 | FHL1 | SBDS | GTF2I | PRKCB | PECAM1 | ZC3H12A | TEX264 |
| SLC31A2 | CH17-340M24.3 | MTRNR2L12 | MBD4 | KMO | FAM127B | CRYAB | ZNF706 |
| ARPC3 | TP53I11 | APOC3 | PCMTD1 | CH17-373J23.1 | TRIB2 | CHPF | GSN |
| LILRA5 | IGLC3 | GNB2L1 | GRINA | RNF149 | PAPSS2 | MFSD12 | MAPK6 |
| SYK | ANGPTL4 | HNMT | LDLRAD4 | FCGR2B | SHE | LIMCH1 | ATP6V1A |
| CCR1 | S100A13 | APOA2 | LSM4 | LRRK2 | ZNF22 | AKAP9 | SNRNP70 |
| RILPL2 | SATB1 | ACTN4 | NDUFB5 | ZNF267 | ARGLU1 | PNRC1 | OCIAD1 |
| RPS24 | OTUD6B-AS1 | CD52 | YTHDF3 | NUP214 | AKTIP | CDC42 | FGB |
| IL10 | DKK3 | NNMT | SURF4 | CAV2 | MT1A | GPR4 | DPY30 |
| NAIP | CTSF | FXYD6 | LUZP1 | P2RY6 | NOP58 | DOCK9 | TOR1AIP2 |
| HCLS1 | CFAP36 | WWTR1 | BLOC1S6 | S100A10 | HIST1H4C | CDH5 | GTF3A |
| RPS2 | MAP4 | RGS16 | UXT | TPM3 | ACTA2 | SEC11C | ZSWIM6 |
| GPNMB | A4GALT | PTP4A2 | LRP10 | MALAT1 | LPP | EVL | APOB |
| CCL3L3 | FZD4 | EMCN | ANKRD28 | RPL35 | NASP | AK3 | PDCD5 |
| UBA52 | PLA2G5 | SEC62 | ECI2 | IFNGR1 | TSPAN7 | RAP1A | CALHM2 |
| TNNI2 | PPP1CA | EHD2 | EHMT1 | SLC16A3 | LEPROT | MT1X | HNRNPA3 |
| FCGRT | YES1 | B2M | EI24 | IGFBP4 | PGRMC2 | HSP90B1 | NANS |
| CD48 | RPS27L | MZB1 | C21orf59 | ARPC2 | TSPAN3 | CXorf21 | TMEM173 |
| EMILIN2 | PTN | AKR1B1 | GZF1 | SDS | CX3CL1 | PARVA | NT5C |
| MMP9 | MACF1 | HYAL2 | SLC38A2 | BCAM | FYN | WT1-AS | NRIP1 |
| RAB7B | AGT | ENO1 | LINC01485 | INSIG1 | CPPED1 | PBXIP1 | CUX1 |
| EVI2B | TALDO1 | LPCAT1 | HSPA2 | ARL4A | CLN8 | PCAT19 | OAT |
| HACD4 | CDC42SE2 | RNH1 | PPP1R16A | TNFAIP3 | CNKSR3 | TRMT1 | PIK3R1 |
| DOK2 | NUDT4 | VEGFA | MAP2K3 | ARID5B | MGLL | IGHGP | EIF5B |
| LAT2 | CLDN5 | JAG1 | ISCA1 | EPAS1 | LAMA5 | AKR1A1 | CHCHD6 |
| HLA-DQB2 | CYB5R3 | EZR | PGM2L1 | AKIRIN2 | KMT2A | RPL23A | BCLAF1 |
| HLA-DQA2 | IPO11 | SOCS2 | KIAA0040 | LIPA | KLF13 | ZBTB38 | CHMP5 |
| WDFY4 | PAM | FN1 | ARL5B | RPL8 | MTRNR2L1 | PROS1 | DCTN3 |
| CD4 | PTPN18 | HEXB | TRBC2 | PRDX2 | BCL2L1 | PRELID1 | NDUFS3 |
| CTSH | ICAM2 | RAMP2 | MRFAP1 | COL4A1 | IQGAP1 | LIMA1 | GC |
| RTN1 | IGLV3-1 | MCAM | ALDH1A1 | FXYD5 | SHC1 | HLA-DOB | MPC2 |
| CTSZ | ATP5J2 | TGFB1I1 | SUMO3 | MRC1 | MOCS2 | TMEM263 | HSPA6 |
| PARVG | FNBP1L | UACA | IGLC2 | RIN3 | CDH13 | CDC42EP1 | APOE |
| CACNA2D3 | SRPRA | DOCK5 | EMC4 | GPR157 | CCDC80 | RPS4X | ORM1 |
| CHMP1B | PLTP | ARHGDIB | SERINC3 | SPCS2 | SCO2 | ATP6AP2 | TMEM107 |
| COTL1 | IFITM1 | GRB2 | TMEM255B | CD302 | SPOCK2 | ATP5L | PARL |
| SH3BGRL3 | EPN2 | PDE4DIP | GINM1 | ADRBK2 | TPR | RPL35A | PTPN1 |
| CORO1A | NEXN | DLC1 | ITIH3 | SERPINH1 | IGHG3 | EPHX1 | APOC2 |
| EVI2A | LTBP4 | EDNRB | NDUFC1 | ADAM28 | HOPX | SMOC2 | FGA |
| CECR1 | TMEM223 | BEST1 | DCP2 | SYNE2 | CXorf36 | STAB1 | GEM |
| GPSM3 | APBB2 | GADD45A | ADAM17 | KDM6B | ECM2 | GPX3 | NDUFC2 |
| PPIF | RHOA | KLF4 | CHST12 | DSTN | EMC7 | CAPZA1 | APOH |
| CCRL2 | SYNPO2 | RPS10 | C3orf58 | SPRY1 | WDR60 | LMCD1 | CREG1 |
| CD44 | PLAU | ATP13A3 | MGMT | HSPB1 | ATP2B4 | LRRC8A | TF |
| H2AFY | TPMT | IGFBP5 | TCEAL8 | ARHGAP30 | TRBC1 | HMHA1 | CES1 |
| RPL10 | BTNL9 | FAM46C | PDLIM2 | LILRB1 | HIBCH | FCRL5 | CEP170 |
| CTSB | F10 | RPL6 | KDELR2 | PRR13 | C7orf55 | B3GNT5 | HSPA9 |
| CD53 | ATP5D | IFITM2 | POLR2I | GPX4 | PITPNB | CCND1 | MTRNR2L6 |
| IDO1 | FMO3 | MITF | SCAMP2 | HMGA1 | PGRMC1 | TM4SF18 | KLF2 |
| CD300E | TRMT6 | OCIAD2 | HIF1A | OPTN | TRAPPC4 | RDX | C4BPA |
| TNFAIP8L2 | TBX2 | GSAP | POLR2J3 | SDCBP | MAGEH1 | PTPRB | OAS1 |
| GSTP1 | COLEC11 | ETS1 | APLP2 | TET2 | SGCE | MYC | POLR2K |
| RPL12 | RCN1 | PCDH17 | MRPS18B | COL18A1 | RNF115 | DNAJA1 | PPDPF |
| NR4A3 | HSD17B11 | CLEC14A | ADPGK | PTGS2 | SOX4 | TSPAN12 | TAX1BP3 |
| PABPC1 | FBLIM1 | OSTF1 | BUB3 | TINAGL1 | RNF11 | EMP1 | NDUFA12 |
| RAB31 | GSTA4 | HSPG2 | CCL20 | PLSCR1 | ASPN | XBP1 | QKI |
| IER3 | PGD | RASSF2 | VWF | RGS1 | TTC28 | LAMC1 | SERPIND1 |
| OGFRL1 | RAB30 | APOLD1 | ATP5J | CREM | PLEKHG1 | KCNN3 | SQSTM1 |
| NFKBID | FKBP15 | ID1 | UPP1 | SMS | LYST | SRSF5 | CCDC12 |
| REL | COX6B1 | ZEB1 | RNF19A | ASAH1 | SERPINE2 | TSC22D3 | SSR4 |
| ZEB2 | PCOLCE | CD79A | VCL | SMCO4 | SNRK | RND3 | TMEM109 |
| RPS9 | PLCB1 | TNS2 | CCT3 | PFKFB3 | RHOQ | DLL4 | UGP2 |
| TMSB10 | NOP10 | CD151 | DPH3 | IFI27 | AFAP1L1 | HMGN1 | PLIN2 |
| RPL28 | MLLT4 | IL10RA | BAG3 | MANBA | SH3BP2 | MALT1 | CCL5 |
| CD84 | PLEKHA2 | LAMA4 | BEX4 | MYO9B | UPF3A | TMEM219 | SMCHD1 |
| ACP5 | HEYL | RPS6 | SRSF11 | ARPC4 | CSAD | A2M | HSPH1 |
| JUN | HSPE1 | TANK | MASP2 | DOCK2 | ITIH2 | PTPRK | ROMO1 |
| NAMPT | KCNJ8 | NDUFA4L2 | KMT2E | ARHGAP29 | HEY1 | CTSD | PRAP1 |
| RPS11 | CCDC50 | ALOX5AP | CDKN2C | ID3 | PTGR1 | HEG1 | SPAG9 |
| C19orf38 | H1F0 | DUSP2 | SNX18 | CD59 | AEBP1 | BTF3 | KNG1 |
| MAFB | HCFC1R1 | SORL1 | MAPKAP1 | TNFRSF1B | NFASC | IGHG2 | ARL2 |
| CLECL1 | C1QTNF1 | VIMP | KRT8 | NGFRAP1 | PNISR | HIGD1B | C8G |
| LCP2 | UNC119 | TMEM204 | UBE2J1 | ESAM | OXA1L | TXN |  |

**Supplementary Table S6. List of 127 intersecting genes.**

| ALOX5 | COL6A1 | RASSF2 | GGT5 | LGALS3 | COLEC11 | SMOC2 | FHL2 |
| --- | --- | --- | --- | --- | --- | --- | --- |
| FCER1A | LRRC32 | ID1 | FOXS1 | PLXDC2 | FBLIM1 | LMCD1 | KIAA0040 |
| RAB31 | NES | LAMA4 | EDNRA | MAP1B | HEYL | RND3 | NCOA7 |
| CAV1 | RCAN2 | NPDC1 | COL5A2 | COL6A2 | C1QTNF1 | HEG1 | SLC12A2 |
| KCTD12 | COL3A1 | PLAT | PMEPA1 | EGR1 | PXDN | SLC44A2 | TPPP3 |
| SPARC | PDLIM3 | MYL9 | MYO10 | TPM1 | GALNT18 | PBX1 | COL1A1 |
| VCAN | TP53I11 | PRSS23 | ACTA2 | LBH | CTGF | TAGLN | DPYSL2 |
| IGFBP7 | DKK3 | DCN | IQGAP1 | THY1 | PDGFD | CRISPLD2 | PDLIM7 |
| ARID5B | A4GALT | ISYNA1 | CCDC80 | MFGE8 | AIF1L | SOD3 | PLXND1 |
| COL4A1 | PLA2G5 | AQP1 | ASPN | PPP1R14A | COL14A1 | F2R | THBS1 |
| TINAGL1 | PTN | FRZB | SERPINE2 | BGN | ARHGEF17 | FSTL1 | FSCN1 |
| ID3 | PAM | PGF | AEBP1 | ANXA1 | AKT3 | C11orf96 | CALHM2 |
| COL4A2 | NEXN | PDGFRB | LOXL2 | EHD2 | MMP2 | EFEMP2 | TMEM173 |
| CYR61 | LTBP4 | NOTCH3 | PDGFA | JAG1 | PTPRN2 | GLO1 | GEM |
| MGP | PLAU | MSRB3 | CSPG4 | MCAM | ELK3 | PROCR | KLF2 |
| METRNL | TBX2 | COL1A2 | FOS | IGFBP5 | FBN1 | MYH11 |  |

**Supplementary Table S7. Results of univariate Cox regression analysis.**

| Gene | HR (95%CI) | P-value |
| --- | --- | --- |
| PDLIM3 | 1.137 (1.019–1.270) | 0.022 |
| PAM | 1.065 (1.011–1.121) | 0.017 |
| PDLIM7 | 1.039 (1.009–1.069) | 0.010 |
| FSCN1 | 1.007 (1.002–1.012) | 0.009 |
| DPYSL2 | 1.042 (1.007–1.077) | 0.017 |
| ARID5B | 1.114 (1.024–1.212) | 0.012 |
| LGALS3 | 1.006 (1.002–1.011) | 0.008 |
| CSPG4 | 1.077 (1.007–1.151) | 0.030 |
| KLF2 | 0.951 (0.907–0.996) | 0.032 |
